# The pan-cancer landscape of prognostic germline variants in 10,582 patients

**DOI:** 10.1186/s13073-020-0718-7

**Published:** 2020-02-17

**Authors:** Ajay Chatrath, Roza Przanowska, Shashi Kiran, Zhangli Su, Shekhar Saha, Briana Wilson, Takaaki Tsunematsu, Ji-Hye Ahn, Kyung Yong Lee, Teressa Paulsen, Ewelina Sobierajska, Manjari Kiran, Xiwei Tang, Tianxi Li, Pankaj Kumar, Aakrosh Ratan, Anindya Dutta

**Affiliations:** 1 Department of Biochemistry and Molecular Genetics, University of Virginia School of Medicine, 1240 Pinn Hall, Charlottesville, VA 22908 USA; 2 Department of Systems and Computational Biology, School of Life Sciences, University of Hyderabad, Hyderabad, Telangana India; 3 Department of Statistics, University of Virginia, Charlottesville, VA USA; 4 Center for Public Health Genomics, University of Virginia, Charlottesville, VA USA

**Keywords:** Germline variants, Single nucleotide polymorphism, Cancer biology, Pan-cancer, Survival analysis, Tumor suppressor, Oncogene, Driver gene, Non-synonymous mutation, eQTL

## Abstract

**Background:**

While clinical factors such as age, grade, stage, and histological subtype provide physicians with information about patient prognosis, genomic data can further improve these predictions. Previous studies have shown that germline variants in known cancer driver genes are predictive of patient outcome, but no study has systematically analyzed multiple cancers in an unbiased way to identify genetic loci that can improve patient outcome predictions made using clinical factors.

**Methods:**

We analyzed sequencing data from the over 10,000 cancer patients available through The Cancer Genome Atlas to identify germline variants associated with patient outcome using multivariate Cox regression models.

**Results:**

We identified 79 prognostic germline variants in individual cancers and 112 prognostic germline variants in groups of cancers. The germline variants identified in individual cancers provide additional predictive power about patient outcomes beyond clinical information currently in use and may therefore augment clinical decisions based on expected tumor aggressiveness. Molecularly, at least 12 of the germline variants are likely associated with patient outcome through perturbation of protein structure and at least five through association with gene expression differences. Almost half of these germline variants are in previously reported tumor suppressors, oncogenes or cancer driver genes with the other half pointing to genomic loci that should be further investigated for their roles in cancers.

**Conclusions:**

Germline variants are predictive of outcome in cancer patients and specific germline variants can improve patient outcome predictions beyond predictions made using clinical factors alone. The germline variants also implicate new means by which known oncogenes, tumor suppressor genes, and driver genes are perturbed in cancer and suggest roles in cancer for other genes that have not been extensively studied in oncology. Further studies in other cancer cohorts are necessary to confirm that germline variation is associated with outcome in cancer patients as this is a proof-of-principle study.

**Electronic supplementary material:**

The online version of this article (10.1186/s13073-020-0718-7) contains supplementary material, which is available to authorized users.

## Background

Large-scale sequencing projects increased our molecular understanding of cancers to the point where using sequencing data to augment clinical decisions seems promising [1, 2]. Somatic mutations in cancers have received substantial attention in oncology as they can be used to individualize drug selection [2, 3]. While much effort has been directed towards characterizing somatic mutations in cancer, recent studies suggest that germline variants also have significant clinical utility.

In line with the heritability of some cancers, several germline variants predict a patient’s risk for developing cancer and are useful for individualizing cancer screening guidelines [4–13]. Germline variation can affect drug sensitivity, predict drug toxicity, and could help select therapy to minimize side-effects [14–26]. Some germline variants increase patient risk for specific somatic aberrations, suggesting that germline variation may impact disease course [27].

We hypothesized that the effects of germline variants on cancer progression may be strong enough to identify associations with patient outcome. Previous studies tested for an association between patient outcome and a small number of germline variants in genes well-characterized in a given cancer [28, 29]. We published an unbiased method of testing for an association between a large number of germline variants and patient outcome in patients with lower-grade gliomas [30]. In this study, we identify prognostic germline variants using sequencing data from 10,582 patients from The Cancer Genome Atlas (TCGA). These germline variants significantly improve predictions of patient outcome compared to clinical variables alone, identify biological mechanisms by which germline variants affect patient outcomes, and identify genes and pathways that impact cancer biology and therapy.

## Methods

### Data sources, variant calling, and quality control

The results in this manuscript are based upon data generated by The Cancer Genome Atlas (TCGA) Research Network: https://www.cancer.gov/tcga. We determined the germline variant statuses of 10,582 cancer patients by variant calling the patients’ whole-exome sequenced normal samples (WXS normal), whole-exome sequenced tumor samples (WXS tumor), and RNA sequenced tumor samples (RNA tumor) available on Cancer Genomics Cloud using VarDict (mapping quality > 30, base quality > 25, variant reads > 2, minimum allele frequency > 5%, no duplicate reads) and determined the sequencing depth at each position using samtools (mapping quality > 30) [31–33]. We set variant calls to unknown if the position at which the variant was called was covered by fewer than 10 reads. We then merged these three variant call sets, giving preference to WXS normal then WXS tumor then RNA tumor. We only included variants with an allele frequency of greater than 5% in the non-Finnish European population of gnomAD, variants found in more than 14 patients in a given cancer, and variants whose calls were greater than 90% concordant with each other in a given cancer in our final analysis [34]. These thresholds had been selected in our previous study in order to better tune the allele frequencies of the European patients in our study to previously reported population frequencies [30]. Our quality control tests for setting these thresholds yielded similar results across the other cancers outside of the lower-grade gliomas. We labeled variant calls as concordant for a given variant if they gave the exact same variant call (homozygous for the reference allele, heterozygous, or homozygous for the alternate allele) in the WXS normal, WXS tumor, and RNA tumor samples. Variant calls were therefore discordant if the variant call differed in any of the three samples. The percentage concordance was calculated for each germline variant by dividing the total number of concordant variant calls by the total number of patients and multiplying the result by 100%.

We retrieved clinical outcome data for each patient using the TCGA Pan-Cancer clinical data resource [35]. We used TCGAbiolinks to obtain patient clinical information, and we downloaded patient race composition from The Cancer Genome Ancestry Atlas (TCGAA) [36, 37]. Additional clinical information for the lower-grade glioma and glioblastoma patients was downloaded from a previous analysis [38]. We used Lasso regularization to determine which clinical covariates should be controlled for in our models, while using patient race composition from TCGAA in place of patient-reported race [39, 40]. The patient race composition reported in the TCGAA more accurately captured the genetic ancestry of the TCGA patients compared to patient-reported race as patient race composition is quantitative and multidimensional. Where we did not control for patient race composition in cancers where patient race composition was not identified as a significant predictor of patient outcome by Lasso-regularized Cox regression, we later retested the set of prognostic germline variants by adding back patient race composition as a covariate into our Cox regression models. As expected, because patient race composition was not a significant predictor of patient outcome in these cancers, we still found all of our originally identified prognostic germline variants to be statistically significant predictors of patient outcome. We also found that the hazard ratios estimated in the original models (without race) with the retested models (with race) were highly correlated (Spearman rho = 0.983, *p* = 7.63E−47).

We were not able to control for treatment. As discussed in greater detail by Liu et al., it is very difficult to control for treatment in the TCGA dataset [35]. Detailed treatment information was not submitted in a consistent manner for many of the patients in TCGA and absence of submitted treatment information does not necessarily mean that the patient did not receive treatment. Furthermore, treatment regimens are quite complex and depend on chemotherapy drug selection and dosage, extent of surgical excision, and radiation therapy, among other factors. The broad spectrum of treatment options makes treatment challenging to control for. As discussed by Liu et al., the TCGA treatment information will likely need to be evaluated by panels of cancer specialists before it can be used for modeling in pan-cancer studies [35]. Nevertheless, it is unlikely that differences in treatment accounted for the bulk of the associations observed in this study. The most natural way for treatment differences to account for the observation that germline variation is associated with patient outcome is due to socioeconomic differences associated with patient race or unconscious or conscious biases in treatment selection based on patient race. However, we accounted for calculated genetic ancestry as part of our pipeline, making these possibilities unlikely.

We determined the number of somatic mutations in the cancer samples and evaluated the overlap between germline variants and somatic mutations and RNA editing sites as previously described [30]. To ensure that our variant calls from the four variant call sets (WXS normal, WXS tumor, RNA tumor, and Combined) were concordant with each other, we calculated the allele frequency of each variant as in our previous analysis and calculated the Spearman correlation coefficient of these allele frequencies with each other.

### Power analysis

We performed a power analysis in individual cancers to evaluate our ability to detect associations between germline variants and patient outcome using Cox regression. The power to detect an association between a germline variant and patient outcome is dependent on the sample size, effect size, correlation with other covariates in the model, the number of individuals with the germline variants, and the number of individuals without a germline variant, among other factors. As a result, the power to detect an association differs between germline variants, even assuming the same hazard ratio. To estimate our power, we therefore randomly sampled 10,000 germline for each cancer from the pool of germline variants to be tested in that cancer. We calculated statistical power using the powerSurvEpi R package (https://cran.r-project.org/web/packages/powerSurvEpi/index.html). We calculated our power to detect a significant association at a significance level (α) of:

$$ \frac{0.10}{\mathrm{Total}\ \mathrm{number}\ \mathrm{of}\ \mathrm{germline}\ \mathrm{variants}\ \mathrm{tested}\ \mathrm{in}\ \mathrm{that}\ \mathrm{cancer}} $$

This threshold would be as stringent or slightly more stringent than false discovery correction using the Benjamini-Hochberg procedure which we ultimately used in our analysis. We then calculated the percentage of germline variants for which we had greater than 80% statistical power to detect a significant association at hazard ratios of 2, 3, 4, 5, 10, 15, and 20.

### Identification of prognostic germline variants

We utilized six total approaches for identifying prognostic germline variants. In all analyses, we tested variants for an association with outcome using a Cox regression model, controlling for the covariates that we identified previously for each cancer using Lasso regularization. We used the R packages survminer (https://cran.r-project.org/web/packages/survminer/index.html) and survival (https://cran.r-project.org/web/packages/survival/index.html) to perform Cox regression and generate Kaplan-Meier plots. *p* values were corrected for multiple hypothesis testing using the Benjamini-Hochberg procedure. The circos plots were generated using the R package circlize [41].

In analysis 1, we tested variants for an association with patient outcome in individual cancers, setting an adjusted *p* value threshold (FDR) less than 0.10. We reported all statistically significant results and did not filter our results based on a hazard ratio threshold, as it is difficult to know what hazard ratio threshold would be clinically and biologically relevant. In the second analysis, we filtered our results from analysis 1 to identify germline variants that were recurrently associated (*p* < 0.05) with favorable (hazard ratio (HR) < 1) or poor (HR > 1) outcome relative to the reference allele in seven or more cancers, such that the most recurrent prognostic variants would be reported. Given that molecular similarities between some of the TCGA cancers may have made it more likely that certain germline variants would be picked up in this second analysis than others, we did not think that it would be statistically valid to estimate the probability of variants being pulled out by this analysis by chance. In the third analysis, we grouped the cancers based on clinical understanding about the cancers and clustering patterns observed previously by the TCGA research network [42]. We tested germline variants for associations with patient outcome (FDR < 0.10) in these larger groups to detect germline variants with smaller effect sizes. In pooling cancers, we implicitly assumed that the germline variant had similar effects in the grouped cancers. If this assumption was not true for a particular germline variant, then that germline variant would actually be less likely to be associated with patient outcome. Only variants found in 15 or more patients across all grouped cancers were tested, resulting in fewer variants being tested in this analysis.

Analyses 4–6 were quite similar to analyses 1 through 3, except that we restricted our analysis to only germline variants that caused significant amino acid changes with a Combined Annotation Dependent Depletion (CADD) score greater than 25 [43]. This enabled us to identify associations that we did not capture in analyses 1 through 3 due to the relatively higher stringency in that analysis resulting from multiple hypothesis correction. In analysis 4, we tested variants with CADD score > 25 in individual cancers for an association with patient outcome (FDR < 0.10). In analysis 5, we filtered the results from analysis 4 to identify germline variants with CADD score > 25 that were recurrently associated (*p* < 0.05) with favorable (HR < 1) or poor (HR > 1) prognosis in five or more patients. In analysis 6, we tested germline variants with CADD > 25 for a significant association (FDR < 0.10) with patient outcome in the previously described patient groups.

The Cox regression models that we fit for individual cancers controlled for the covariates that we found to be prognostic in those cancers (Additional file 1: Table S1). The Cox regression models that we fit for patient groups controlled for the covariates that we found to be prognostic in individual cancers with each term containing an interaction term associating that variable with the cancer that it was associated with patient outcome in. We also controlled for cancer type in these combined groups. As an example, suppose that variable A is associated with patient outcome in cancer X and variable B is associated with patient outcome in cancer Y. Then we would fit two Cox regression models to identify prognostic germline variants in individual cancers and a third Cox regression model to identify germline variants prognostic in the pooled cohort, as illustrated below.

1. Identifying germline variants associated with patient outcome in cancer X
  $$ \mathrm{Patient}\ \mathrm{outcome}\sim {\beta}_0+{\beta}_1\left(\mathrm{variable}\ \mathrm{A}\right)+{\beta}_2\left(\mathrm{germline}\ \mathrm{variant}\ \mathrm{status}\right) $$
2. Identifying germline variants associated with patient outcome in cancer Y
  $$ \mathrm{Patient}\ \mathrm{outcome}\sim {\beta}_0+{\beta}_1\left(\mathrm{variable}\ \mathrm{B}\right)+{\beta}_2\left(\mathrm{germline}\ \mathrm{variant}\ \mathrm{status}\right) $$
3. Identifying germline variants associated with patient outcome when the patients with cancer X and the patients with cancer Y are pooled together
  $$ \mathrm{Patient}\ \mathrm{outcome}\sim {\beta}_0+{\beta}_1\left(\mathrm{cancer}\ \mathrm{X}\ \mathrm{status}\right)+{\beta}_2\left(\mathrm{cancer}\ \mathrm{X}\ \mathrm{status}\left)\right(\mathrm{variable}\ \mathrm{A}\right)+{\beta}_3\left(\mathrm{cancer}\ \mathrm{Y}\ \mathrm{status}\right)\left(\mathrm{variable}\ \mathrm{B}\right)+{\beta}_4\left(\mathrm{germline}\ \mathrm{variant}\ \mathrm{status}\right) $$

In model (3) above, cancer X status is a dummy variable that can be 0 or 1. The value of this variable is 0 for patients with cancer Y and 1 for patients with cancer X. The opposite is true for the cancer Y status variable. This allowed us to group patients to test for an association with patient outcome, while controlling for differences between different cancers and relevant clinical differences between patients with the same cancer.

### Concordance and correlation of hazard ratios for the prognostic germline variants

We tested whether germline variants associated with patient outcome (*p* < 0.05) in three of more cancers were typically recurrently associated with increased risk of poor outcome or recurrently associated with decreased risk of poor outcome more often than would be expected by random chance and if the hazard ratios estimated for these prognostic germline variants in different cancers were correlated with each other.

To test for concordance, we first counted the number of times that germline variant was found to be associated (*p* < 0.05) with poor patient outcome (HR < 1) or favorable patient outcome (HR > 1). We then calculated the following value for each prognostic germline variant:

$$ \frac{\max \left(\mathrm{poor}\ \mathrm{outcome},\mathrm{favorable}\ \mathrm{outcome}\right)}{\mathrm{poor}\ \mathrm{outcome}+\mathrm{favorable}\ \mathrm{outcome}} $$

where poor outcome is the number of times that the germline variant was associated with poor outcome (HR < 1) and favorable outcome is the number of times that the germline variant was associated with favorable outcome (HR > 1). If a germline variant was perfectly concordant, then the calculated value would be 1. While theoretically the expected value would be 0.5 for a random germline variant, we empirically estimated the expected value by the following calculation:

$$ \frac{\max \left(\mathrm{total}\ \mathrm{number}\ \mathrm{of}\ \mathrm{poor}\ \mathrm{outcome},\mathrm{total}\ \mathrm{number}\ \mathrm{of}\ \mathrm{favorable}\ \mathrm{outcome}\right)}{\mathrm{total}\ \mathrm{number}\ \mathrm{of}\ \mathrm{poor}\ \mathrm{outcome}+\mathrm{total}\ \mathrm{number}\ \mathrm{of}\ \mathrm{favorable}\ \mathrm{outcome}} $$

In this set of prognostic variants, there were more variants associated with poor patient outcome (HR < 1) than favorable patient outcome (HR > 1), resulting in the expected index being 0.589. We then used a Wilcoxon rank sum test to determine whether the concordance values that we calculated from the set of prognostic germline variants differed from what we would expect by random chance.

We next tested whether the hazard ratios estimated for a given prognostic germline variant in different cancers were correlated with each other. Because we had previously found the hazard ratios to be concordant, we performed this analysis separately for instances in which a germline variant was found to be associated with increased risk of poor outcome and decreased risk of poor outcome. We identified the set of variants associated with favorable (HR < 1) outcome and poor (HR > 1) outcome in three or more cancers. The set of variants that were associated with favorable and poor outcome were analyzed separately. For each analysis, we generated all possible pairs of hazard ratios for a given germline variant. We then ran a Spearman’s correlation test to determine whether or not the hazard ratios were correlated to each other. Because the hazard ratio is also correlated to the allele frequency, we repeated the prior analysis with a Spearman partial correlation test to control for germline variant allele frequency. Partial correlation was calculated used the ppcor R package [44].

### Characteristics of prognostic germline variants

Having identified the prognostic germline variants, we then aimed to compare the characteristics of prognostic germline variants to the characteristics of germline variants identified in previous genome-wide association studies [45]. We decided to use the variants from analysis 1 and analysis 3 to understand the characteristics of prognostic germline variants because the other approaches each identified a very small number of prognostic germline variants. We decided not to pool all of the germline variants together due to possible differences in characteristics between these sets of variants. We therefore analyzed the characteristics of the prognostic germline variants from analysis 1 and from analysis 3 separately. To avoid considering the same information multiple times, we removed variants that were linked with each other from the analyses in this section and only retained the first variant by genomic position. The actual variant retained did not have a significant effect on our results because the hazard ratios and sample sizes for the linked variants were very similar.

We first tested whether or not the minor allele was typically associated with poor patient outcomes. We sorted the variants into two categories: minor alleles that were associated with poor outcome in the Cox regression model (HR > 1) and minor alleles that were associated with favorable outcomes (HR < 1). Although the reference allele was often the major allele, this was not always the case. We performed a one-sided Fisher’s exact test in R to determine whether or not the minor allele was more likely to be associated with poor outcome. The R package scatterpie (https://cran.r-project.org/web/packages/scatterpie/index.html) was used to display the proportion of homozygous reference, heterozygous, and homozygous alternate individuals. For variants in analysis 3 that were pulled out in multiple groups, we displayed the proportion of individuals only for the group that contained the largest number of individuals. The largest group always contained all individuals because the smaller groups were made up of smaller number of cancers and was always contained in the larger group. For example, suppose a variant was found to be prognostic in both group 20 (KICH, KIRP) and group 19 (KICH, KIRC, KIRP). In this case, we would perform all calculations using the information from group 19.

We next tested whether or not there was an inverse correlation between effect size and allele frequency. To do this, we calculated the Spearman correlation coefficient between effect size, calculated as ∣ ln(HR) − 0 ∣ , and allele frequency. Finally, we identified the genomic regions (upstream of a gene, 5′ UTR, exonic, intronic, 3′ UTR, downstream of a gene, or intergenic) in which each variant was located in using annovar [46]. Some variants were found in multiple different transcripts and therefore mapped to several different genic regions. For the purposes of creating the figures, we allowed a single variant to count once for multiple different regions. Excluding these variants from the figures did not change our interpretation of the results.

### Testing whether the effects of the prognostic germline variants are at least partially independent

If the effects of the prognostic germline variants are at least partially independent of each other, we would expect that if two prognostic germline variants are found in the same patient that the outcome observed in those patients would be even more extreme than the outcome in patients with only a single germline variant. In other words, a patient with two prognostic germline variants associated increased risk for poor outcome should have a worse outcome than a patient with only one prognostic germline variant associated with poor outcome.

To test this hypothesis, we analyzed the set of prognostic variants identified in individual cancers. We set a few boundaries on our analysis to reduce bias.

1. We identified prognostic germline variants highly linked to each other and only kept the first prognostic germline variant by chromosomal position in this set. The determination of which germline variant was selected did not substantially alter our results.
2. We analyzed pairs of variants in individual cancers. Although we could evaluate multiple prognostic variants in each of the cancers, this would make the analysis more complex, given the differing effect sizes of the prognostic germline variants.
3. Because most of the prognostic variants in individual cancers were associated with increased risk for poor outcome, we limited this analysis to only variants associated with increased risk for poor outcome and excluded variants associated with favorable outcome.
4. In the testing of each pair of prognostic germline variants, we excluded individuals who were homozygous for one of the prognostic germline variants. Our Kaplan-Meier plots suggest that for some of the prognostic germline variants, having two copies of the variant has a stronger effect than having a single copy, so including homozygotes for the prognostic germline variants could confound our results. The homozygotes for the prognostic germline variant were relatively rare and so we could not test them separately. Since they were relatively rare, the exclusion of homozygotes for the prognostic germline variant did not dramatically reduce our sample size.

Having setup the conditions for this test, we created three groups for each pair of prognostic germline variants associated with poor patient outcome:

1. Patients homozygous for the reference allele of both prognostic germline variants
2. Patients heterozygous for one of the two prognostic germline variants and homozygous for the reference allele of the other prognostic germline variant
3. Patients heterozygous for both of the prognostic germline variants

We then tested for differences in patient outcome between groups (2) and (1) and groups (3) and (1). If the effects of the prognostic germline variants are at least partially independent, we would expect the hazard ratio from the comparison of groups (3) and (1) to be greater than the hazard ratio from the comparison of groups (2) and (1). We calculated these hazard ratios for each pair of prognostic germline variants and ran a paired one-sided Wilcoxon signed-rank test to evaluate whether the hazard ratio from the comparison of groups (3) and (1) was greater than the hazard ratio from the comparison of groups (2).

### Association of prognostic germline variants with somatic driver mutations

We tested whether the prognostic germline variants were more likely to be associated with somatic mutations in driver genes than would be expected by random chance. We retrieved the set of driver genes for each cancer and consensus somatic mutation calls for each cancer from TCGA Network analyses [2, 47]. For each cancer, we only considered driver genes with five or more patients with a somatic mutation in that driver gene in that cancer. For each prognostic germline variant, we tested whether the variant associated with increased risk of poor outcome was associated with an increased incidence of somatic mutations in each of the driver genes being considered for that cancer in patients with the allele associated with increased risk of poor outcome compared to patients with the protective allele using one-sided Fisher’s exact test. *p* values were adjusted using the Benjamini-Hochberg procedure.

We were then able to determine the number of germline variants that were associated with a somatic mutation in a driver gene. We repeated this approach for all germline variants included in this analysis and performed one-sided Fisher’s exact test to determine whether or not more prognostic germline variants than expected were associated with a somatic mutation in a driver gene.

### Area under the curve

To assess the clinical relevance of our findings, we tested whether the germline variants enhanced patient outcome predictions made using clinical information alone. While we had identified germline variants associated with outcome controlling for clinical covariates, we aimed to determine whether these variants significantly improved patient outcome predictions beyond predictions made using the clinical model alone, particularly in cancers in which the prediction by the clinical model was already quite accurate. We generated receiver operator characteristic (ROC) curves from the tenth percentile of patient death or patient progression to the ninetieth percentile of patient death or patient progression for each variant in R (https://cran.r-project.org/web/packages/survivalROC/survivalROC.pdf, https://cran.r-project.org/web/packages/timeROC/timeROC.pdf). We generated two ROC curves per variant: (1) the first was made using only patient clinical information (C) and (2) the second was generated using both patient clinical information and germline variant status (C + GV). We ran a one-sided Wilcoxon rank sum test in R to determine whether the model supplemented with germline variant status consistently yielded better predictions across time for each variant. While our Cox regression analysis identified variants that were significantly associated with patient outcome, these variants may not necessarily substantially improve clinical outcome predictions in cancers in which the clinical variables are already very good at predicting outcome. Running the one-sided Wilcoxon rank sum test allowed us to test whether the improvement to the prediction was significant.

### Gene annotation and literature review

We annotated the variants resulting from our analysis using biomaRt [48, 49]. We reviewed the literature for the functions of these genes to understand their functions. Many of the authors (RP, SK, ZS, SS, BW, TT, JA, KL, TP, ES, MK) initially reviewed the literature for information about each gene. The literature review was then verified by three of the authors (RP, SK, ZS) to ensure consistency and validity.

Having generated a list of genes that the germline variants are associated with from biomaRt, we first specifically searched the literature to see if these genes had a function in cancer that had been characterized and that fit a category described by Weinberg and Hanahan [50]. This part of the literature review had the largest number of unknowns due to the large amount of specificity required by the studies. We then relaxed our stringency and checked to see whether or not the gene was associated with findings in the literature consistent with oncogenic or tumor suppressor activity in the context of cancer. The classification of the genes as oncogenes or tumor suppressors was based on published biochemical or molecular studies of the genes in the context of cancer. Multiple studies supported the classification as either an oncogene or tumor suppressor gene for a substantial number of the genes. Finally, to understand in general whether or not these genes are being actively studied by the field, we categorized these genes based on whether or not the literature suggested that the genes are being studied in a cancer in which the germline variant was found to be prognostic, studied in any cancer, or studied in any human disease. We also overlapped our gene list with the list of driver genes generated by the TCGA research network [2].

### Variant mechanisms and literature review

We next aimed to understand the mechanisms by which the prognostic germline variants may be exerting their effects. We started with the germline variants that were predicted to cause significant amino acid changes (CADD > 25). We determined the position and amino acid change caused by these germline variants using Ensembl [51]. We determined the domain in which these germline variants cause their amino acid changes using the National Center for Biotechnology Information databases (https://www.ncbi.nlm.nih.gov/) and the Ensembl and Uniprot databases [52]. We next identified germline variants that are likely acting as expression quantitative trait loci in *cis* (*cis* eQTLs). For each germline variant, we separated patients based on whether or not they had at least one non-reference allele and then determined whether or not there was a statistically significant difference between the mean expression of the gene associated with the variant between the two groups using a Wilcoxon rank sum test. We then combined our prediction as to whether the germline variant was protective or associated with increased risk of poor outcome with the expression difference between the two groups to determine whether increased expression of the gene would be expected to be protective or associated with increased risk of poor outcome. We fit Cox regression models using the expression of each of the genes, controlling for clinical covariates, and compared the result to our prediction. We reported variants that are concordant with our predictions. Because the differential expression and Cox regression results had to both be concordant with each other, we used a more relaxed cut-off of *p* < 0.10 for hypothesis generation. Further studies with larger cohorts and statistically more power are necessary to further interrogate these associations. Finally, we checked to see whether the eQTL was also reported in GTEx in the tissue from which the tumor was derived by downloading the list of tissue-specific and pan-tissue eQTLs and comparing the eQTLs identified in our analysis to those reported in GTEx.

We reviewed the literature for previous associations tied to these variants reported in the literature. As was the case with gene annotation, the literature review was first done by multiple authors (RP, SK, ZS, SS, BW, TT, JA, KL, TP, ES, MK) with the final round of quality control and verification being done by a single author (BW).

### Correlation with drug sensitivity

We found the germline variant rs1800932 in *MSH6* to be associated with favorable patient outcome and increased *MSH6* expression. Because a previous analysis found that *MSH6* knockdown resulted in increased temozolomide resistance, we tested whether *MSH6* expression was correlated with temozolomide sensitivity in cancer cell lines [53]. To do this, we downloaded *MSH6* expression levels and temozolomide sensitivity for 915 cell lines using data from the Genomics of Drug Sensitivity in Cancer database through CellMinerCDB [54, 55]. We tested for an association using Spearman’s correlation test.

### Pathway dysregulation

For selected prognostic germline variants described in the text, we tested whether or not these prognostic germline variants were associated with upregulation or downregulation of genes in specific pathways. For each prognostic germline variant, we separated patients into two groups based on whether or not the variant allele was called in those patients. We calculated the log fold change of each gene expressed greater than a median of 1 fragment per kilobase per million mapped reads and used these values as an input for gene set enrichment analysis [56].

## Results

### Identification of high-quality germline variants

Germline variants were called and filtered as shown in Additional file 1: Figure S1 using sequencing data from 10,582 TCGA patients with 33 different types of cancers. In total, 77.6 million unique variants were called. After filtering, we limited our analysis to 519,319 unique variants (Additional file 1: Figure S2). Because the final variant call set was created by merging variant calls from whole-exome sequenced (WXS) normal tissue samples, WXS tumor samples, and RNA sequenced tumor samples, we evaluated our variant calls for contamination by somatic mutations or RNA editing. Our final germline variant call set did not substantially overlap with somatic mutations or RNA editing sites (Additional file 1: Figure S3–S4, Additional file 1: Text S1).

### Determination of prognostic clinical models for each cancer

To identify prognostic germline variants that provide additional outcome information not already captured by clinical variables, we created clinical models predictive of patient outcome for each cancer using the clinical information previously collected by the TCGA research network along with the components of calculated race from The Cancer Genome Ancestry Atlas. The variables selected for each cancer are summarized in Additional file 1: Table S1. The study was powered to capture prognostic germline variants with moderate to high effect sizes (beginning at hazard ratios > 2) (Additional file 1: Figure S5, Additional file 1: Text S2).

### Identification of prognostic germline variants

The 191 prognostic germline variants from the six analyses are described in Additional file 2: Table S2A-F.

**Fig. 1:**
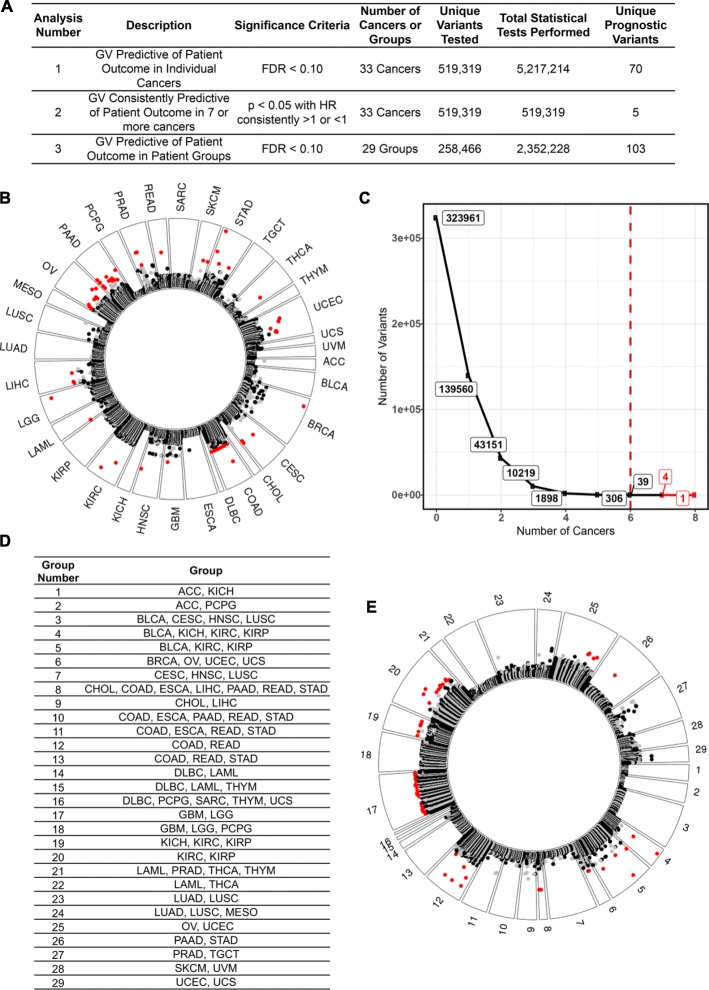
Prognostic germline variants identified in analyses 1 through 3. **a** A description of the three analyses used to identify prognostic germline variants in this figure. **b** Analysis 1. Germline variants found to be predictive of patient outcome in each cancer. Each dot represents a germline variant that was tested for an association with patient outcome. Variants closer to the outside of the plot are more closely associated with patient outcome. Variants in red are significantly (FDR < 0.10) associated with patient outcome. The alternating black and gray colors reflect alternating chromosomes for the germline variants that were not significant predictors of patient outcome. **c** Analysis 2. Germline variants found to be recurrently predictive of patient outcome in multiple different cancers. We identified five total germline variants that were recurrently predictive (*p* < 0.05) of favorable (HR < 1) or poor (HR > 1) patient outcomes in seven or more different cancers. **d** Analysis 3. A total of 29 groups of cancers created to identify germline variants with weaker effect sizes in larger patient cohorts. Justification for these groups is provided in Additional file 1: Table S3. **e** Analysis 3. Germline variants found to be predictive of patient outcome in the groups described in Fig. 1d. The format of the figure is the same as in Fig. 1b.

**Fig. 2:**
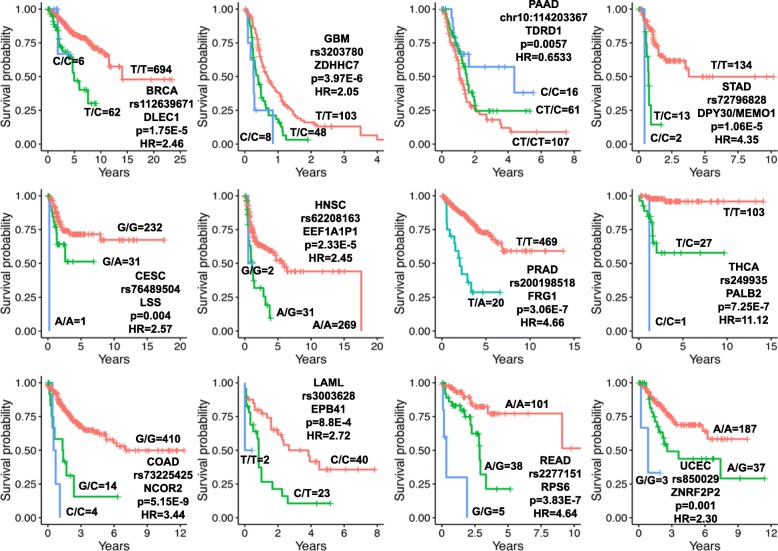
Selected Kaplan-Meier plots of the prognostic germline variants from analysis 1. The number of patients in each group is indicated next to each line, and the patient outcome measure of each disease is given in Additional file 1: Table S1. The reported *p* values and hazard ratios were calculated using univariate regression and are different from the *p* values and hazard ratios reported elsewhere which are based on multivariate regression

The first three analyses identified germline variants associated with prognosis in (1) individual cancers, (2) multiple cancers giving roughly equal weight to each cancer, and (3) cancers grouped by organ system, histological, or molecular classifications (Fig. 1a). Analysis 1 tested 519,139 variants for associations with patient outcome in individual cancers and identified 70 unique prognostic variants (Fig. 1b, Additional file 2: Table S2A, Kaplan-Meier plots of selected examples in Fig. 2).

While analysis 2 identified hundreds of variants recurrently predictive of outcome in > 4 cancers, we will only discuss the 5 variants that were predictive in seven or more cancers (Fig. 1c, Additional file 2: Table S2B). Both the direction of the hazard ratios (increased or decreased risk of poor outcome) and the magnitude of the effect on patient outcome for germline variants across different cancers were highly correlated (Additional file 1: Text S3).

Analysis 3 increased our statistical power by grouping similar cancer types to increase the number of patients with the minor allele that could be included in the study. A total of 29 different patient groups were created based on the organ system, histological, or molecular classification (Fig. 1d, group justification in Additional file 1: Table S3). In total, 258,466 unique germline variants were tested and 103 prognostic variants were identified (Fig. 1e, Additional file 2: Table S2C, Kaplan-Meier plots of selected examples in Additional file 1: Figure S6).

### Prognostic germline variants causing significant amino acid changes

**Fig. 3:**
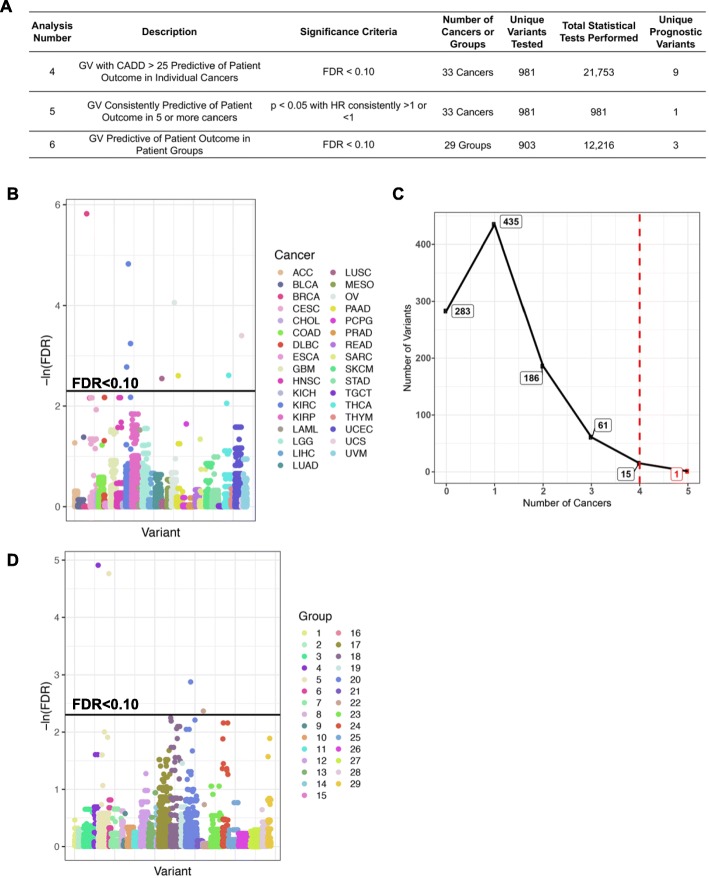
Prognostic germline variants that cause significant amino acid changes (CADD > 25) identified in analyses 4 through 6. **a** A description of the three analyses used to identify prognostic germline variants in this figure. **b** Analysis 4. Germline variants causing significant amino acid changes found to be predictive (FDR < 0.10) of patient outcome in each cancer. **c** Analysis 5. Germline variants causing significant amino acid changes found to be recurrently predictive (*p* < 0.05) of favorable (HR < 1) or poor (HR > 1) patient outcomes in 5 or more different cancers. **d** Analysis 6. Germline variants causing significant amino acid changes found to be predictive of patient outcome in patient groups defined in Fig. 1d

Analyses 4–6 repeated analyses 1–3 but limited these analyses to variants within the top 0.3% of deleterious mutants across the human genome with CADD > 25 (Fig. 3a). Analysis 4 tested a total of 981 unique variants and identified nine unique prognostic variants (Fig. 3b, Additional file 2: Table S2D). Of the 16 variants that were recurrently predictive of patient outcomes in four or more cancers (analysis 5), we will discuss the one variant that was predictive in five cancers (Fig. 3c, Additional file 2: Table S2E). Analysis 6 tested 903 unique variants for an association with outcome in the patient groups used in analysis 3 and described in Fig. 1d and identified 3 additional prognostic variants (Fig. 3d, Additional file 2: Table S2F).

### The pan-cancer landscape of prognostic germline variants

The large number of prognostic variants identified in analyses 1 and 3 allowed us to compare the characteristics of these germline variants with previously reported characteristics of variants identified by genome wide association studies (GWAS). Three characteristics have been noted in variants identified through GWAS: (1) the minor allele tends to be associated with increased risk for poor outcome when considering the set of variants with large effect sizes, (2) there is a negative correlation between effect size and allele frequency, and (3) most germline variants identified by GWAS do not cause amino acid changes [45].

**Fig. 4:**
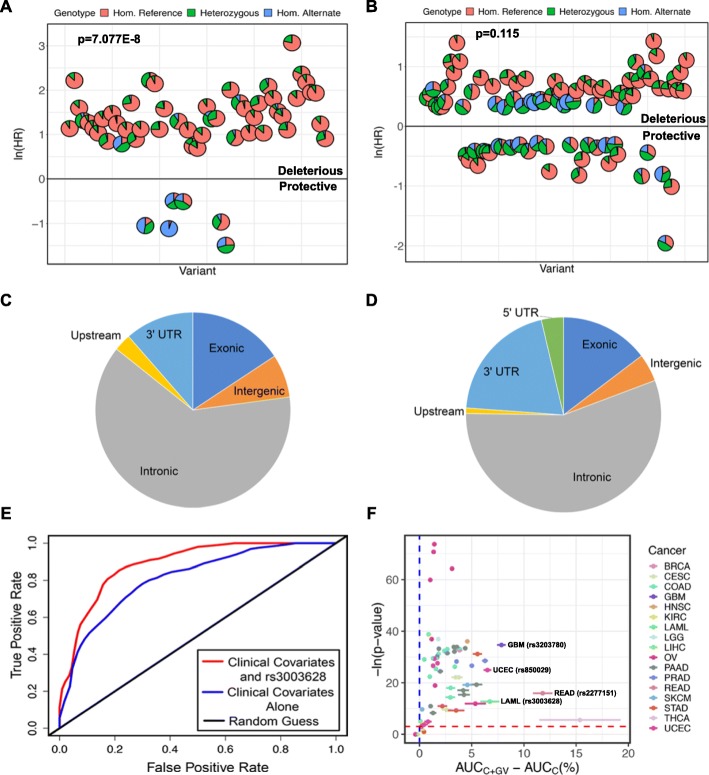
Characteristics of prognostic germline variants and improvement of patient outcome models by the prognostic germline variants. **a, b** Scatterplots of the prognostic germline variants identified in individual cancers in analysis 1 (**a**) and in groups of cancers in analysis 3 (**b**). Each pie chart reflects the distribution of patients that are homozygous for the reference allele, heterozygous, and homozygous for the alternate allele for one prognostic variant. The minor allele was much more likely to be associated with increased risk for poor outcome rather than decreased risk for poor outcome (*p* = 7.077E−8) in analysis 1 though this trend was not significant in analysis 3 (*p* = 0.115). **c, d** Pie charts displaying the genomic locations of the germline variants in analysis 1 (**c**) and analysis 3 (**d**). **e** An example of a receiver operator characteristic (ROC) curve calculated using data from LAML at 366 days of follow-up. The blue line represents the patient outcome predictions made using clinical information alone (C model). The red line represents patient outcome predictions made using clinical information in addition to rs3003628 germline variant status (C + GV model), which we found to be predictive of patient outcomes in LAML. The area under the curve (AUC) was 0.81 for the C model and 0.93 for the C + GV model giving a ΔAUC of 0.12 (12%). **f** Many of the prognostic germline variants improve clinical outcome model predictions. For each prognostic variant, we created a ROC curve based on the clinical (C) model and the clinical + germline variant (C + GV model), as in Fig. 4e, at each point in time from the 10th-90th percentile of patient progression or death for each cancer. The ΔAUC of the C + GV model versus the C model at each time point was calculated (Additional file 3: Table S4). *X*-axis: Mean and standard error of ΔAUC. *Y*-axis: The *p* values from testing whether or not the AUC of the C + GV model is significantly greater than that of the C model using a Wilcoxon rank sum test. Four examples of prognostic germline variants that significantly increase the AUC are labeled and highlighted in Additional file 3: Table S4

To test whether the allele associated with increased risk for poor outcome is usually the minor allele, the predictive alternate alleles from analysis 1 were classified as associated with increased risk for poor outcome (HR > 1) or decreased risk for poor outcome (HR < 1) based on the Cox regression results. Of the prognostic germline variants from analysis 1, the allele associated with increased risk is clearly often the minor allele (*p* = 7.077E−8) (Fig. 4a). A similar analysis with the predictive variants from analysis 3 (Fig. 4b) did not show a significant statistical depletion of alternate alleles associated with increased risk for poor outcome from the population (*p* = 0.115). The predictive variants from analysis 3 were detectable only with larger sample sizes and have smaller effect sizes than those identified by analysis 1. Thus, the result in Fig. 4b is still consistent with the first premise that an allele associated with increased risk for poor outcome with a large effect size (as in analysis 1, but not analysis 3) is usually the minor allele [45].

A negative correlation is seen between effect size and allele frequency with both variants from analysis 1 (Spearman’s rho = − 0.282, *p* = 0.0184) and analysis 3 (Spearman’s rho = − 0.667, *p* < 2.2E−16), satisfying the second premise. Finally, the vast majority of predictive variants identified by this study do not cause amino acid changes (Fig. 4c, d), satisfying the third premise.

If the effects of the prognostic germline variants are at least partially independent of each other, we would expect that patients with two prognostic germline variants that increase the risk for poor outcome should do worse than patients with only one of these prognostic germline variant that increases the risk for poor outcome. Indeed, when tested, we found this to be true (*p* = 8.45E−17, analysis approach detailed in “Methods”).

A previous study had identified germline variants associated with an increased incidence of somatic mutations in cancer-related genes [27]. We also found that some of the prognostic germline variants were associated with an increased risk of somatic mutations in cancer driver genes. While more prognostic germline variants were associated with an increased risk of somatic mutations in driver genes than was expected by random chance (OR = 1.89, *p* = 0.0001, Additional file 1: Text S4), not all of the prognostic germline variants were associated with an increased risk of such somatic mutations. A more detailed study of somatic mutations in driver genes is necessary that will take into account differences in genes and cancer types.

### Germline variants significantly improve outcome prediction models

The effect sizes of prognostic germline variants from analysis 1 were large enough to hypothesize that germline variants identified in individual cancers could improve clinical outcome models in current use.

The clinical variables predictive of outcome (Additional file 1: Table S1) were used to generate the first outcome model (Clinical: C). The second outcome model was based on clinical information plus the status of a particular predictive germline variant (Germline Variant: GV) (C + GV). An example receiver operator characteristic (ROC) curve for predicting LAML patient vital status at 366 days of follow-up is shown using C and C + GV for predictive variant rs3003628 (ROC in Fig. 4e). The area under the ROC curves (AUC) for the C model is 0.807 and for the C + GV model is 0.928. The change in AUC (ΔAUC) for the C + GV model relative to the C model in this example is 0.12 (12%). To ensure that the change in AUC is consistent at different times of follow-up, ΔAUC was calculated from the 10th to the 90th percentile of patient outcome time. The mean and standard error of ΔAUC was plotted against the *p* value of the one-sided test evaluating whether the AUC for C + GV is significantly larger than the AUC for C (Fig. 4f).

This analysis was repeated for all predictive variants. There is a consistent, statistically significant (*p* < 0.05) increase in AUC when the clinical model is enhanced by germline variant information (C + GV) compared to the clinical model alone (C) for 63 of the predictive germline variants out of 70 tested (Additional file 3: Table S4). These results demonstrate that adding predictive germline variants to existing clinical criteria will improve the prediction of outcome of many cancers.

### Prognostic variants in driver genes, oncogenes, and tumor suppressor genes

**Fig. 5:**
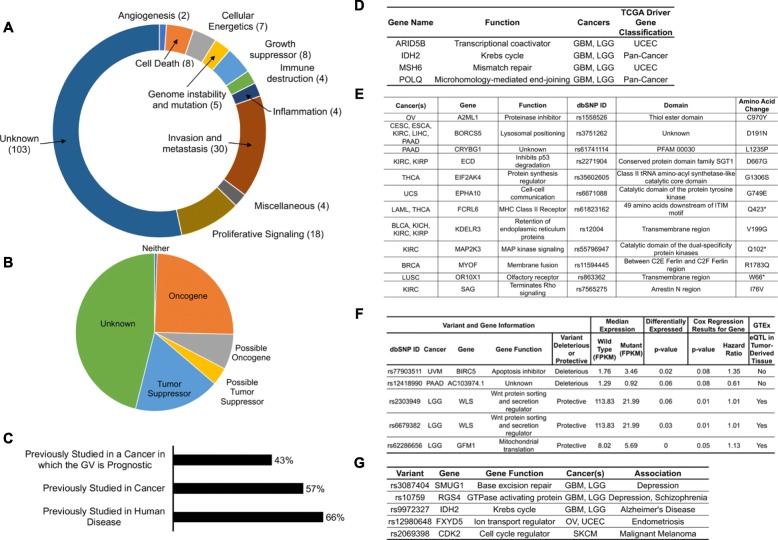
Literature review of genes associated with the prognostic germline variants and mechanisms by which prognostic germline variants may exert their effects. **a** The cancer-related functions of genes associated with the prognostic germline variants are quite diverse. **b** Many of the genes associated with the variants have previously been reported to be tumor suppressor genes or oncogenes. We categorized genes as tumor suppressor genes or oncogenes based on phenotypes reported in the literature, even if the exact mechanism through which the genes act have not yet been determined. **c** Although many of the variants have been studied in the field, there are many genes that have not yet been studied in the context of human disease and therefore may warrant investigation by the field. **d** Four of the genes associated with prognostic germline variants are in previously reported cancer driver genes. **e** Some of the prognostic germline variants cause dramatic amino acid changes and may disrupt well-characterized protein domains. **f** Some of the prognostic germline variants likely act as expression quantitative trait loci in *cis* (*cis* eQTLs) and the expression of these genes are predictive of patient outcome. We found three of these germline variants to also be eQTLs in the genotype tissue expression (GTEx) database in the same tissue that the tumor was derived from. **g** Some of the prognostic germline variants have been reported to be associated with other diseases related to the tissue from which the tumor was derived

In total, 90 of the 193 genes in the proximity of one of the prognostic germline variants have been functionally implicated in nine of the 12 hallmarks of cancer (Fig. 5a, Additional file 4: Table S5) [50].

Roughly 50% of the predictive variants are found in or near genes that possibly have tumor suppressor or oncogenic activity (Fig. 5b, Additional file 4: Table S5). About 25% of the predictive genes were previously studied in the cancer in which the germline variant was found to be prognostic, about half were previously studied in at least one cancer, and roughly two thirds were studied in at least one human disease (Fig. 5c, Additional file 4: Table S5). Prognostic variants were identified in or near *MSH6*, *POLQ*, *ARID5B*, and *IDH2*, which are previously reported cancer driver genes (Fig. 5d).

### Prognostic germline variants can cause significant amino acid changes or act as eQTLs

The 12 prognostic variants identified in analyses 4–6 caused significant amino acid changes (CADD > 25), with many of these amino acid changes occurring in protein-coding domains with annotated or known functions (Fig. 5e).

A total of 39 variants could act as *cis* eQTLs, as they were associated with expression differences of the proximate genes. We highlight five of these variants because the expression levels of the proximate genes are also predictive of survival, with the direction of the effect (HR > 1 or < 1) being concordant with the effect of the variant (Fig. 5f). Of these five variants, three were also *cis* eQTLs in the corresponding tissue in GTEx [57].

### Prognostic variants implicated in other diseases

Some of the prognostic variants are linked with diseases that occur in the tissue giving rise to the tumor, suggesting the variant has an important function in that tissue (Fig. 5g, Additional file 5: Table S6A). Additional file 5: Table S6B lists prognostic genes that are linked in the literature to traits in tissues outside the ones bearing the tumors.

### Individual prognostic variant characterization

In this section, we characterize three germline variants to illustrate how individual germline variants may be associated with patient outcome. These hypotheses are supported by bioinformatic analyses and require future molecular insight to confirm and fully understand the mechanistic underpinnings of these associations.

### rs1800932 in *MSH6* may be associated with favorable outcome by increasing temozolomide sensitivity

rs1800932 predicts favorable patient outcome in gliomas (LGG and GBM). This variant is an eQTL for increased expression of *MSH6* in many tissues, including nerve, is associated with increased expression of *MSH6* in patients with LGG (*p* = 0.00732), and has previously been reported to be associated with a decreased risk of prostate cancer [57, 58]. We found *MSH6* expression to be correlated with elevated temozolomide sensitivity in cancer cell lines (Spearman’s rho = 0.165, *p* = 5.01E−7) [54]. Temozolomide is a DNA alkylating agent used in the treatment of most glioma patients and is likely to have been used in the therapy of most patients with gliomas in TCGA. *MSH6* knockdown increases temozolomide resistance and somatic mutations in *MSH6* are associated with temozolomide resistance in gliomas [53, 59]. Taken together, this suggests that rs1800932 is an eQTL for increased expression of *MSH6* in gliomas, which may increase sensitivity to temozolomide, the primary chemotherapeutic agent for gliomas.

### rs55796947 in *MAP2K3* may result in cell cycle arrest and apoptosis

**Fig. 6:**
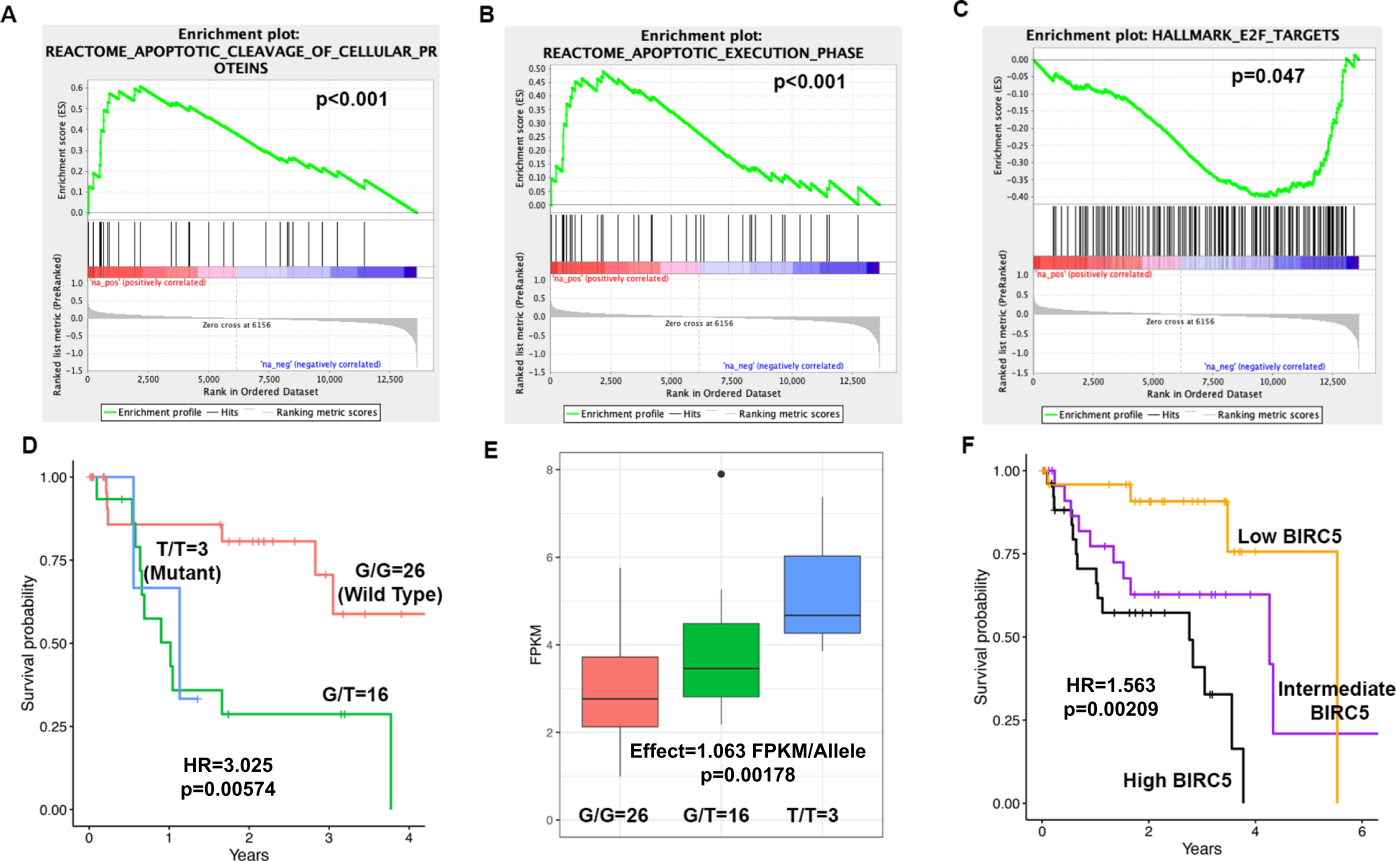
Examples by which two of the prognostic germline variants may be associated with patient outcome. **a–c** rs55796947 in *MAP2K3/MKK3* is associated with favorable patient outcome in KIRC and results in complete loss of *MAP2K3*’s protein kinase domain due to a Q73* amino acid change. *MAP2K3* inhibition has previously been reported to result in cell cycle arrest and response to chemotherapy drugs. Tumors with the variant show upregulation of genes involved with apoptotic cleavage (**a**), genes in the apoptotic execution phase (**b**), and downregulation of E2F targets (**c**) in a Gene Set Enrichment Analysis (GSEA) of RNAseq data. **d–f** rs77903511 in the apoptosis inhibitor *BIRC5* is predictive of poor patient outcome in UVM (**d**). This variant is associated with increased *BIRC5* expression (**e**). Elevated *BIRC5* expression is associated with poor patient outcome (**f**)

rs55796947 in *MAP2K3/MKK3* predicts favorable prognosis in KIRC. This germline variant introduces a stop codon in *MAP2K3* that truncates the kinase domain. *MAP2K3* inhibition results in cell cycle arrest, autophagy-mediated cell death, the unfolded protein response (UPR), and sensitization to chemotherapy drugs [60]. Indeed, tumors in patients with this variant upregulate genes involved with apoptosis (*p* < 0.001, Fig. 6a, b) and downregulate *E2F* targets involved in cell cycle progression (*p* = 0.047, Fig. 6c). This germline variant likely truncates the kinase domain of *MAP2K3*, resulting in cell cycle arrest, apoptosis, and favorable patient outcome.

### rs77903511 is an eQTL for *BIRC5* which inhibits apoptosis

rs77903511 predicts poor patient outcome in UVM (Fig. 6d). *BIRC5* inhibits apoptosis through interaction with and inhibition of caspase 9 and effector caspases. The alternate allele is associated with increased *BIRC5* expression in the tumors (*p* = 0.02, Fig. 6e). Consistent with a role of *BIRC5* in apoptosis inhibition, *BIRC5* expression is associated with poor patient outcome (Fig. 6f). This variant, therefore, may be associated with poor outcome because of an increase of the apoptosis inhibitor *BIRC5*.

## Discussion

This study shows, as a general principle, that germline variants are associated with cancer patient outcome. The prognostic germline variants enhanced patient outcome predictions compared to models based on currently collected clinical data. We envision germline variants providing clinicians with information about a patient as a supplement to reported history, physical exam findings, and imaging and laboratory tests. These predictions will improve over time with the use of more information available in electronic medical records.

The results of this study are most easily applied at the population level to identify groups of patients at increased risk for poor outcome (for example for clinical trials) and for follow-up mechanistic studies on how the variants affect outcome. This study will serve as the basis for future work to apply these findings at the level of individual patients, as a given variant will need to be considered in conjunction with other variants and with clinical factors to calculate expected survival time or time to progression. While we identified a large number of prognostic germline variants in analysis 1, our sample size for this study was relatively modest. The power calculations and the identification of additional prognostic germline variants by grouping similar cancers suggest that more prognostic germline variants will likely emerge as more tumors are sequenced and will further support the notion that germline variation is associated with patient outcome across cancers. Our study of prognostic germline variants was limited to common germline variants (allele frequency > 5% in the population) due to statistical limitations derived from sample size in our ability to study pathogenic and low-frequency germline variants. However, our results imply that these rarer germline variants may have large effect sizes that may make them particularly valuable for improving clinical outcome model predictions. These variants will likely be studied in the future through more complex approaches or in studies of larger cohorts.

Further study is necessary to validate the associations that we identified, as setting the discovery threshold at FDR < 0.10 suggests that some of the associations may have occurred by random chance. The variants identified in analyses 2 and 5 require deeper interrogation, as we were unable to develop an unbiased test to assess the probability of those associations occurring by random chance. While we identified germline variants associated with significant improvements in clinical outcome predictions, further work is necessary to identify situations in which the additional prognostic information would be valuable for treatment decisions or end of life planning.

Given the paucity of studies testing for associations between germline variants and patient outcome in cohorts of cancer patients, we were unsure of the effect sizes that could be expected in this study across the 33 cancers. This uncertainty was further exacerbated by reports of effect sizes being negatively correlated with allele frequency for some traits [45]. The results of this study will provide researchers with a sense for the magnitude of effect sizes that can be expected from germline variants associated with patient outcome along with the relationship between effect size and allele frequency. These results will help better optimize future studies for detecting significant associations.

It is reassuring that a significant fraction of prognostic germline variants are found in or near possible tumor suppressor genes, oncogenes, or known cancer driver genes. The variants in cancer driver genes, *MSH6*, *POLQ*, *ARID5B*, and *IDH2*, warrant further study to determine the mechanism by which these variant affect cancer progression [61]. The 12 germline variants in Fig. 5e that cause substantial amino acid changes are prime candidates for experimental follow-up and are discussed in detail in Additional file 1: Text S5. A handful of the prognostic germline variants have been associated with human disease, some in the same tissue and others in unrelated tissues, suggesting that these pathologies may stem from shared molecular phenomena (Additional file 5: Table S6).

The mechanisms of action of many of the prognostic variants are currently unknown. There are many possibilities by which the variants that do not cause amino acid changes could affect cancer biology [62]. Many variants are likely acting as *trans* eQTLs, which are difficult to study in datasets with relatively small sample sizes. Some of the variants may also be acting as eQTLs in non-tumor cells, such as immune system cells or cells of the vasculature. The already high involvement of tumor suppressor genes, oncogenes, and driver genes among the prognostic germline variants is promising for future study. This report provides basic science researchers with genes and variants that should be studied to better understand the etiology and progression of cancers, while providing clinicians with the potential for better clinical predictions that could be made if germline variants are considered in the context of patient care.

## Conclusions

While the prediction of outcome for patients with cancer is currently based on clinical factors, the analysis of next-generation sequencing data in clinical oncology has suggested that genomic information can further improve these predictions. Previous studies analyzing the usage of genomic information in clinical oncology have focused primarily on somatic aberrations. In this proof-of-principle study, we systematically analyzed sequencing data from 33 different cancers to test whether germline variation could also be used to provide clinicians with information about patient outcome. We identified prognostic germline variants across individual cancers and group of cancers and find that these germline variants provide additional predictive power about patient outcomes beyond the information that can be gathered from clinical factors alone. Mechanistically, 12 of the germline variants seem to be associated with patient outcome through perturbation of protein structure and at least five through association with gene expression differences, though the molecular functions of most of the germline variants are currently unknown. About half of the germline variants are in previously reported tumor suppressor genes, oncogenes, or driver genes with the other half implicating loci that deserve further investigation in oncology. Further studies of germline variation in other cancer cohorts are necessary to confirm that germline variation is associated with patient outcome across cancers.

## Additional files

Additional file 1: Primary Supplemental File containing all Supplemental Figures, **Table S1**, **Table S3**, and all Supplemental Text.

Additional file 2: **Table S2.** A. 70 Prognostic Germline Variants Identified in Analyis 1. B. Five Prognostic Germline Variants Identified in Analysis 2. C. 103 Prognostic Germline Variants Identified in Analysis 3. D. 9 Prognostic Germline Variants Identified in Analysis 4. E. 1 Prognostic Germline Variants Identified in Analysis 5. F. 3 Prognostic Germline Variants Identified in Analyis 6. (XLSX 48 kb)

Additional file 3: **Table S4.** Detailed information about each of the prognostic germline variants and the area under the curve values depicted graphically in Fig. 5f. The examples labeled in Fig. 5f are highlighted in the table below. (XLSX 461 kb)

Additional file 4: **Table S5.** Literature review results for the genes that the prognostic germline variants were found in or near (Fig. 6a–c). (XLSX 26 kb)

Additional file 5: **Table S6.** A. Prognostic germline variants previously found to be associated with a trait related to the tissue from which the tumor was derived (Fig. 5g). B. Prognostic germline variants found to be associated with other traits in the literature outside of the tissue from which the tumor was derived. (XLSX 11 kb)

## Abbreviations

ACC: Adrenocortical carcinoma
AUC: Area under the curve
BLCA: Bladder urothelial carcinoma
BRCA: Breast invasive carcinoma
C: Clinical
CADD: Combined Annotation Dependent Depletion score
CESC: Cervical squamous cell carcinoma and endocervical adenocarcinoma
CHOL: Cholangiocarcinoma
COAD: Colon adenocarcinoma
DLBC: Lymphoid neoplasm diffuse large B cell lymphoma
eQTL: Expression quantitative trait loci
ESCA: Esophageal carcinoma
FDR: False discovery rate
GBM: Glioblastoma multiforme
GTEx: Genotype Tissue Expression Project
GV: Germline variation
GWAS: Genome-wide association study
HNSC: Head and neck squamous cell carcinoma
HR: Hazard ratio
KICH: Kidney chromophobe
KIRC: Kidney renal clear cell carcinoma
KIRP: Kidney renal papillary cell carcinoma
LAML: Acute myeloid leukemia
LGG: Brain lower-grade glioma
LIHC: Liver hepatocellular carcinoma
LUAD: Lung adenocarcinoma
LUSC: Lung squamous cell carcinoma
MESO: Mesothelioma
OV: Ovarian serious cystadenocarcinoma
PAAD: Pancreatic adenocarcinoma
PCPG: Pheochromocytoma and paraganglioma
PRAD: Prostate adenocarcinoma
READ: Rectum adenocarcinoma
RNA Tumor: RNA sequenced tumor sample
ROC: Receiver operator characteristic curve
SARC: Sarcoma
SKCM: Skin cutaneous melanoma
TCGA: The Cancer Genome Atlas
TCGAA: The Cancer Genome Ancestry Atlas
TGCT: Testicular germ cell tumors
THCA: Thyroid carcinoma
THYM: Thymoma
UCEC: Uterine corpus endometrial carcinoma
UCS: Uterine carcinosarcoma
UPR: Unfolded protein response
UVM: Uveam melanoma
WXS: Normal whole-exome sequenced normal sample
WXS Tumor: Whole-exome sequenced tumor sample
WXS: Whole-exome sequencing
