# Supplementary material for: The pan-cancer landscape of prognostic germline variants in 10,582 patients
Source: Genome Med. 2020 Feb 17;12:15. doi: 10.1186/s13073-020-0718-7 (PMC7027124; doi:10.1186/s13073-020-0718-7)
Supplement: Supplementary file 1 — Primary Supplemental File containing all Supplemental Figures, Table S1, Table S3, and all Supplemental Text. [file 13073_2020_718_MOESM1_ESM.docx]

**Additional File 1: Supplementary Figures, Tables, Text, and References**

**Table of Contents**

1. **Table of Contents (Page 1)**
2. **Supplementary Figures (Pages 1-8)**
   1. **Figure S1 (Page 2)**
   2. **Figure S2 (Page 3)**
   3. **Figure S3 (Page 4)**
   4. **Figure S4 (Page 5)**
   5. **Figure S5 (Page 6)**
   6. **Figure S6 (Page 7)**
   7. **Figure S7 (Page 8)**
3. **Supplementary Tables (Pages 9-11)**
   1. **Table S1 (Pages 9-10)**
   2. **Table S2 (Supplemental Excel Table – Additional File 2)**
   3. **Table S3 (Page 11)**
   4. **Table S4 (Supplemental Excel Table – Additional File 3)**
   5. **Table S5 (Supplemental Excel Table – Additional File 4)**
   6. **Table S6 (Supplemental Excel Table – Additional File 5)**
4. **Supplementary Text (12-20)**
   1. **Text S1 (Pages 12-14)**
   2. **Text S2 (Page 15)**
   3. **Text S3 (Page 16)**
   4. **Text S4 (Page 17)**
   5. **Text S5 (Page 18-20)**
5. **Supplementary References (Page 21-22)**

**Supplementary Figures**

**Figure S1.** An overview of our approach to identifying prognostic germline variants. Whole exome sequenced normal (WXS Normal), whole exome sequenced tumor (WXS Tumor), and RNA sequenced tumor (RNA Tumor) samples from 10,582 cancer patients from The Cancer Genome Atlas (TCGA) were variant called. The three variant call sets were merged to create a single Combined variant call set that was used in the rest of the analysis. The variants were filtered to include only common variants that were concordant between the three sequencing datasets. We tested variants for an association with patient outcomes while controlling for clinical covariates using Cox regression models.

**
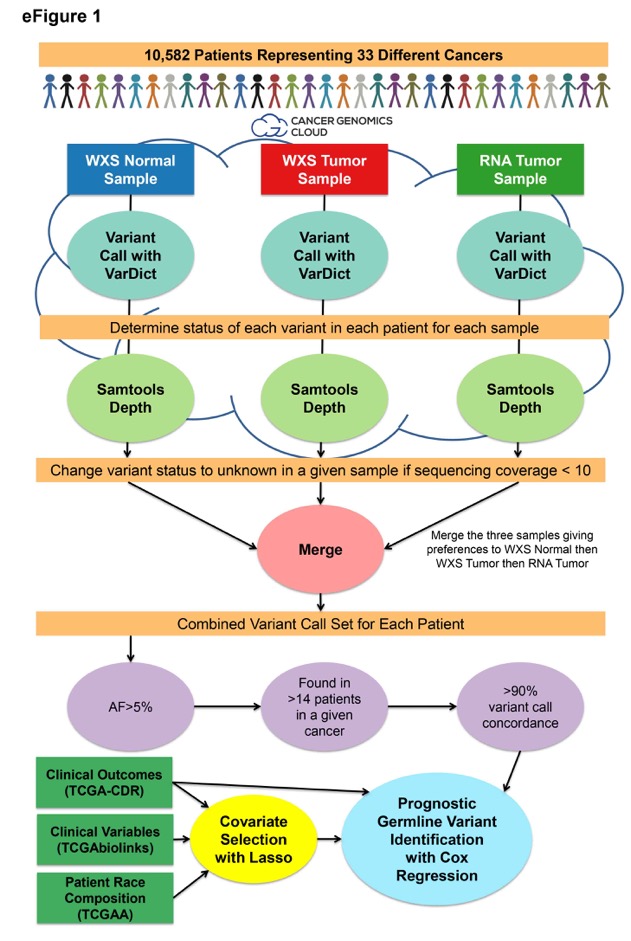
**

**Figure S2**. An overview of the total number of germline variants called and removed by the various filters included in this analysis. 519,319 germline variants were analyzed in this study.


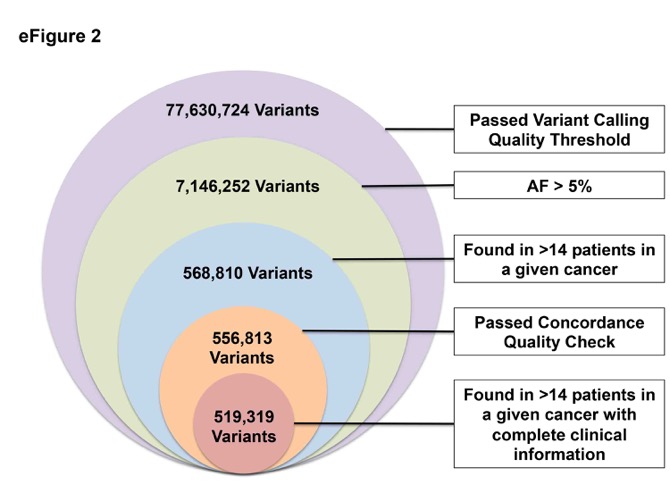


**Figure S3.** Somatic mutations did not compromise the integrity of this study.

**A.** Most variants called from the tumor samples were germline variants. We plotted the percentage of variants called in the whole exome sequenced tumor (WXST) sample that were somatic mutations (SM) across all cancers.

**B.** Few germline variants (GV) cause the same base change as a somatic mutation (SM) across all the cancers after filtering.

**C.** Few germline variants (GV) included in this analysis overlap in genomic position with a somatic mutation (SM).


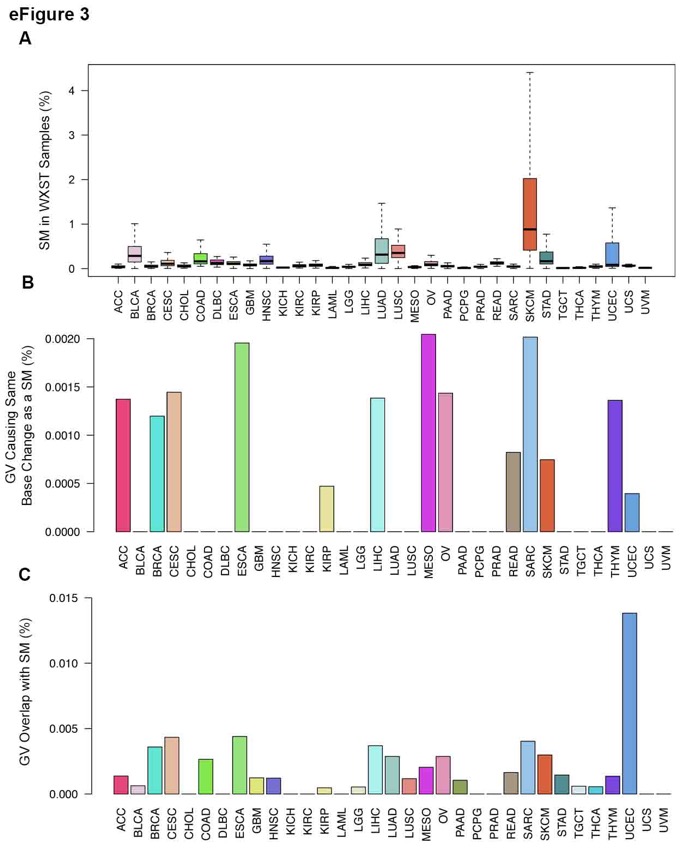


**Figure S4.** RNA editing did not affect the integrity of this analysis.

**A.** Few germline variants (GV) included in this study overlap with a known RNA editing site in genomic position.

**B.** Most germline variants are called in the whole exome sequenced samples (WXS). A relatively small number of germline variants were called solely from the RNA sequenced tumor (RNAT) sample.

**C.** The variant calls from the whole exome sequenced normal (WXSN), whole exome sequenced tumor (WXST), RNA sequenced tumor (RNAT), and Combined (the three variant call sets merged together) are highly concordant with each other. We calculated the allele frequency of each variant in each variant call set and calculated the Spearman correlation coefficient between all pairs.

**
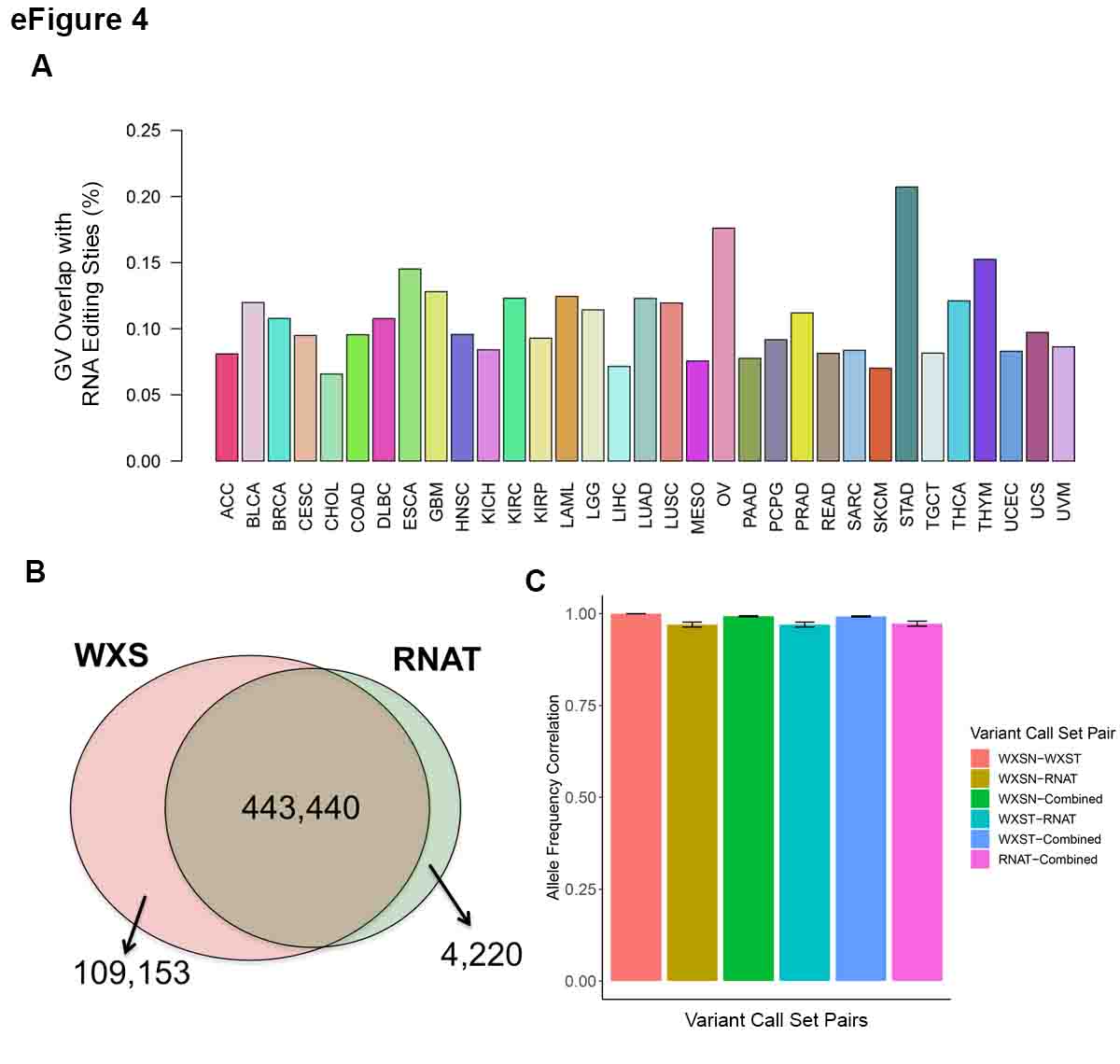
**

**Figure S5.** Power analysis results depicting the percentage of germline variants with >80% power to detect an association between variant status and patient outcome in individual cancers assuming varying effect sizes. To estimate our statistical power, we randomly sampled 10,000 germline variants in each cancer in each iteration and calculated our statistical power to detect an association between each germline variant and patient outcome. The results of this analysis separated the cancers out into three groups:

1. Associations detectable at hazard ratios of moderate magnitudes of 2-3 (BLCA, BRCA, GBM, HNSC, KIRC, LGG, LUAD, LUSC, OV, SKCM, STAD, CESC, COAD, ESCA, LAML, LIHC, MESO, PAAD, PRAD, SARC, THCA, and UCEC)
2. Associations detectable at hazard ratios of moderately high magnitudes of 4-5 (ACC, KIRP, READ, TGCT, UCS, PCPG, THYM, and UVM)
3. Associations detectable at hazard ratios of high magnitudes (CHOL, DLBC, and KICH)

**
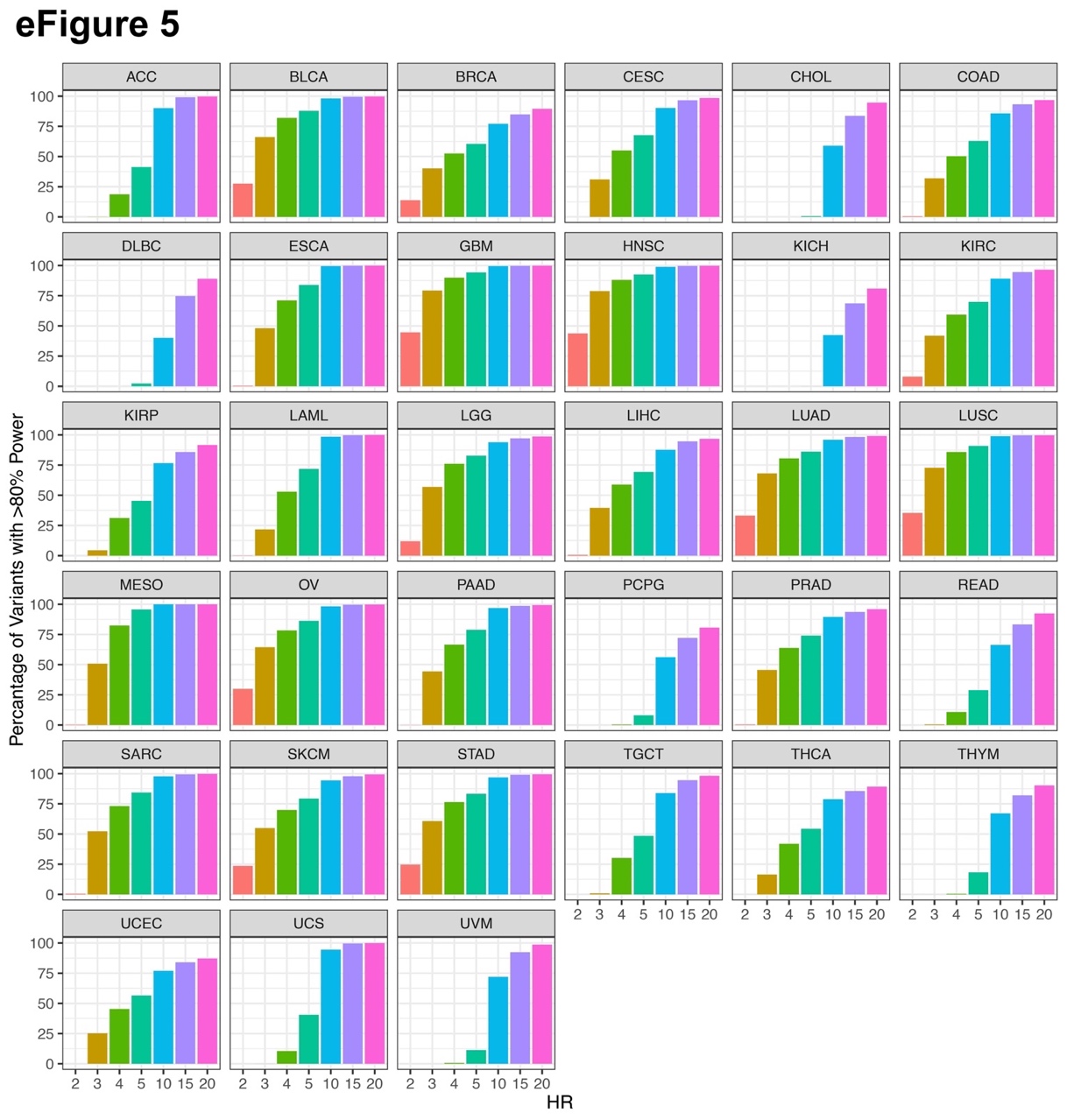
**

**Figure S6.** Selected Kaplan-Meier curves from the variants identified in Analysis 3 in which related cancers were grouped together prior to testing for association with survival.

**
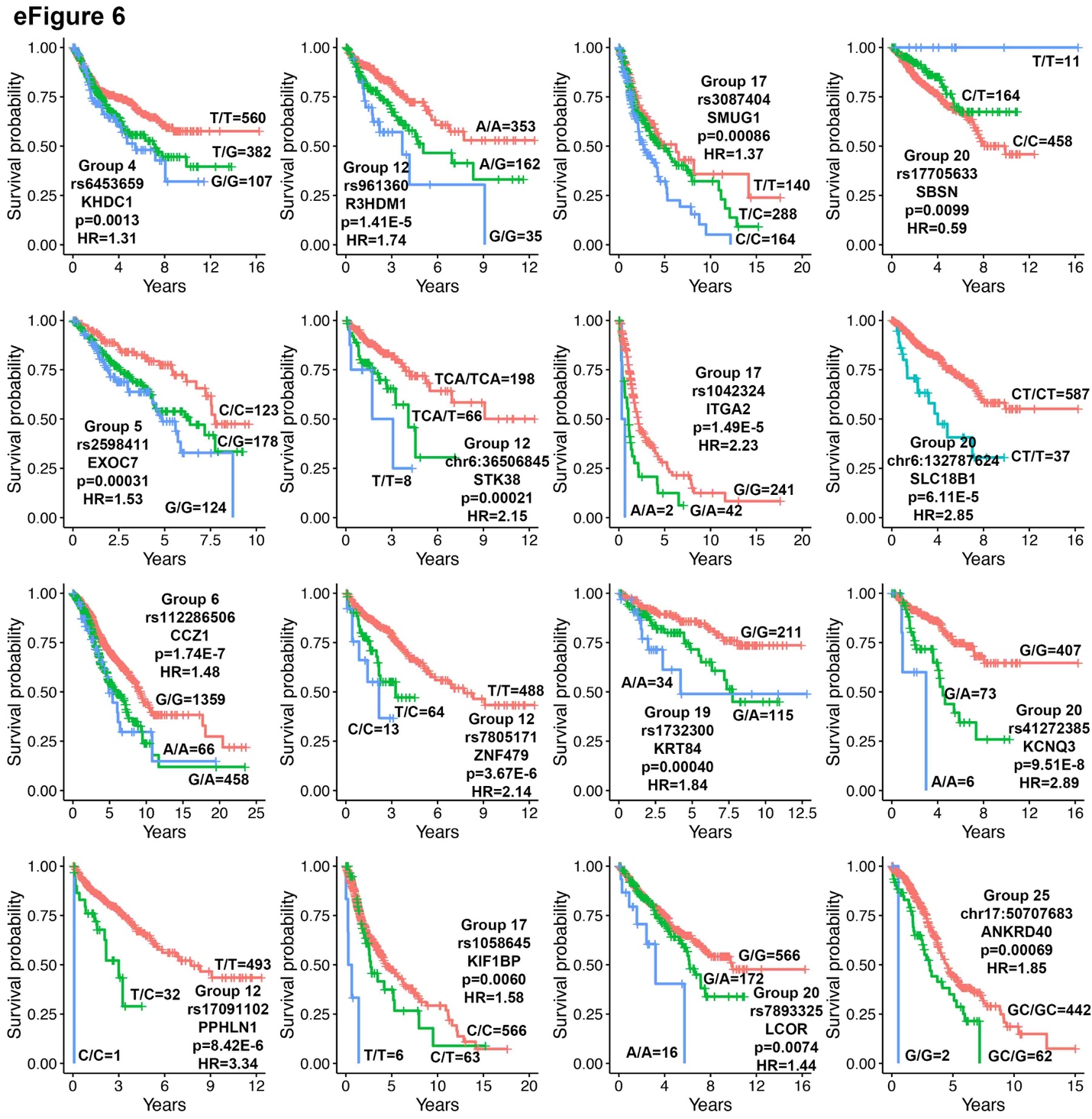
**

**Figure S7.** Schematic representations of how rs1558526, rs6174114, and rs35602605 may perturb well characterize protein domains.

**A.** rs1558526 is associated with favorable patient outcome in OV in the secreted protease inhibitor *A2ML1*. Wild type *A2ML1* inhibits proteases by forming a covalent bond following cleavage of its central bait domain (left). C970 facilitates the formation of this covalent bond. rs1558526 causes a C970Y amino acid change that likely disrupts *A2ML1*’s ability to inhibit proteases (right).

**B.** rs6174114 in *CRYBG1/AIM1* is associated with poor patient outcome in PAAD. The binding of *CRYBG1* to actin requires its 12 βγ crystallin motifs and results in suppression of pro-invasion phenotypes. rs6174114 causes a L1235P amino acid change in the fifth βγ crystallin motifs that may disrupt the packing of the beta sheets and perturb *CRYBG1*’s function, likely leading to increased tumor invasiveness and poor patient outcome.

**C**. rs35602605 in *EIF2AK4/GCN2* is associated with poor prognosis in THCA. *EIF2AK4* decreases translation of some proteins and increases translation of others (such as *CDKN1A*) under conditions of stress by binding uncharged tRNAs through its histidyl-tRNA-synthetase domain. rs35602605 results in a G1306S amino acid change in the histidyl-tRNA synthetase-like domain. This variant may disrupt the function of *EIF2AK4* resulting in poor patient outcome.

**
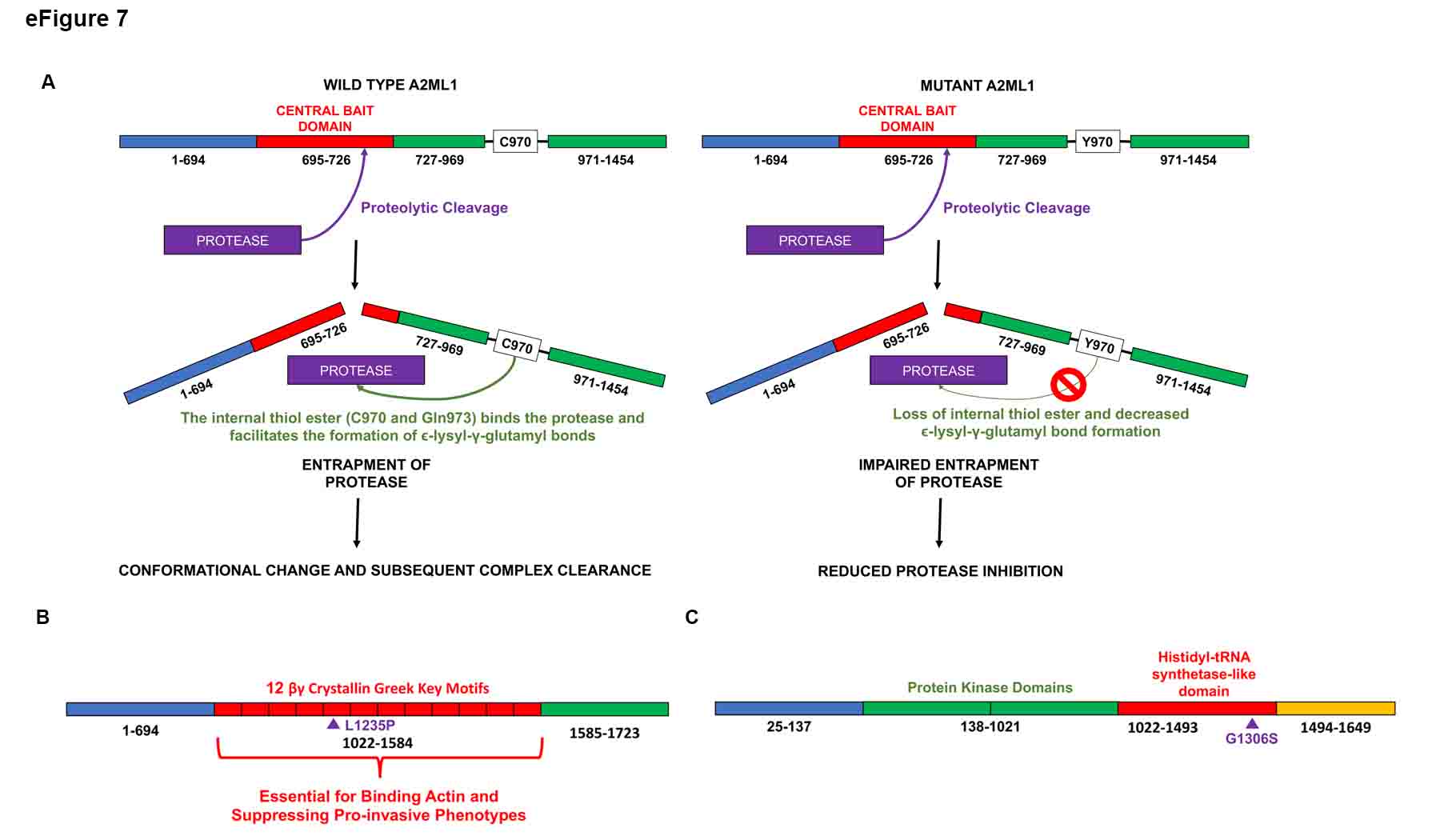
**

**Supplementary Tables**

**Table S1.** Clinical information about the patients included in this study and the covariates that we controlled for in our Cox regression models that were selected using Lasso-regularization.

| **Abbreviation** | **Cancer** | **Sample Size** | **Endpoint** | **Covariates** |
| --- | --- | --- | --- | --- |
| ACC | Adrenocortical carcinoma | 91 | OS | Age, Gender, Calculated Race, Stage |
| BLCA | Bladder Urothelial Carcinoma | 410 | OS | Age, Height, Stage |
| BRCA | Breast invasive carcinoma | 1079 | OS | Age, Estrogen Receptor Status |
| CESC | Cervical squamous cell carcinoma and endocervical adenocarcinoma | 294 | OS | Age, Histological Type, Calculated Race, Stage |
| CHOL | Cholangiocarcinoma | 45 | OS | Albumin Level, Calculated Race |
| COAD | Colon adenocarcinoma | 441 | OS | Age, Anatomic Position, Calculated Race, Stage |
| DLBC | Lymphoid Neoplasm Diffuse Large B-cell Lymphoma | 47 | PFI | None |
| ESCA | Esophageal carcinoma | 184 | PFI | Histological Type, Anatomic Location, Weight |
| GBM | Glioblastoma multiforme | 390 | OS | Age, Chr 19/20 co-gain, Gender, IDH Mutation Status |
| HNSC | Head and Neck squamous cell carcinoma | 523 | OS | Age, Anatomic Location, Grade, Calculated Race, Stage |
| KICH | Kidney Chromophobe | 65 | PFI | Age, Stage |
| KIRC | Kidney renal clear cell carcinoma | 530 | OS | Age, Gender, Grade, Hemoglobin Level, Platelet Count, Calculated Race, Stage, White Blood Cell Count |
| KIRP | Kidney renal papillary cell carcinoma | 286 | OS | Stage |
| LAML | Acute Myeloid Leukemia | 131 | OS | Age, Cytogenetics Risk, Morphology |
| LGG | Brain Lower Grade Glioma | 510 | OS | 1p/19q co-deletion status, Age, Chr 7 gain/Chr 10 Loss Status, Grade, IDH Mutation Status |
| LIHC | Liver hepatocellular carcinoma | 369 | OS | Age, Alcohol Consumption History, Fetoprotein Value, Grade, Platelet Count, Calculated Race, Stage |
| LUAD | Lung adenocarcinoma | 506 | OS | Stage |
| LUSC | Lung squamous cell carcinoma | 497 | OS | Age, Anatomic Location, Calculated Race |
| MESO | Mesothelioma | 85 | OS | Age, Histological Type |
| OV | Ovarian serous cystadenocarcinoma | 523 | OS | Age, Anatomic Location, Grade, Calculated Race, Stage |
| PAAD | Pancreatic adenocarcinoma | 184 | OS | Age, Anatomic Location, Gender, Grade, Calculated Race, Smoking History, Stage |
| PCPG | Pheochromocytoma and Paraganglioma | 177 | PFI | None |
| PRAD | Prostate adenocarcinoma | 498 | PFI | Anatomic Location, Gleason Grade, Calculated Race |
| READ | Rectum adenocarcinoma | 163 | PFI | Age, Gender, Calculated Race, Stage |
| SARC | Sarcoma | 260 | OS | Age, Pathology Margin Status, Postoperative Treatment, Residual Tumor |
| SKCM | Skin Cutaneous Melanoma | 437 | OS | Age, Breslow Depth Value, Calculated Race, Stage |
| STAD | Stomach adenocarcinoma | 416 | OS | Age, Anatomic Location, Grade, Stage, Calculated Race |
| TGCT | Testicular Germ Cell Tumors | 134 | PFI | Anatomic Location, History of Undescended Testis, Calculated Race, Stage |
| THCA | Thyroid carcinoma | 505 | PFI | Histological Type, Stage |
| THYM | Thymoma | 122 | PFI | None |
| UCEC | Uterine Corpus Endometrial Carcinoma | 544 | OS | Age, Grade, Height, Histological Type, Menopausal Status, Calculated Race, Stage, Total Pelvic Lymph Node Ratio, Total Pelvic Lymph Nodes Positive, Weight |
| UCS | Uterine Carcinosarcoma | 56 | OS | Hypertension, Residual Tumor, Total Pelvic Lymph Node Ratio, Tumor Invasion on Primary Pathology |
| UVM | Uveal Melanoma | 80 | OS | Age, Morphology, Tumor Diameter, Year of Diagnosis |

**Table S3**. Justification for the groups presented in **Figure 1D**.

| \| **Group Number** \| **Group** \| **Group Description** \| \| --- \| --- \| --- \| \| 1 \| ACC, KICH \| Clustered by TCGA \| \| 2 \| ACC, PCPG \| Adrenal Tumors \| \| 3 \| BLCA, CESC, HNSC, LUSC \| Clustered by TCGA \| \| 4 \| BLCA, KICH, KIRC, KIRP \| Urinary System \| \| 5 \| BLCA, KIRC, KIRP \| Urinary System Without KICH \| \| 6 \| BRCA, OV, UCEC, UCS \| Female Reproductive \| \| 7 \| CESC, HNSC, LUSC \| Clustered by TCGA \| \| 8 \| CHOL, COAD, ESCA, LIHC, PAAD, READ, STAD \| Gastro-intestinal \| \| 9 \| CHOL, LIHC \| Bile Production and Storage \| \| 10 \| COAD, ESCA, PAAD, READ, STAD \| Digestive System \| \| 11 \| COAD, ESCA, READ, STAD \| Gastro-intestinal Tract \| \| 12 \| COAD, READ \| Colon \| \| 13 \| COAD, READ, STAD \| Lower Gastro-intestinal Tract \| \| 14 \| DLBC, LAML \| Blood \| \| 15 \| DLBC, LAML, THYM \| Immune System \| \| 16 \| DLBC, PCPG, SARC, THYM, UCS \| Clustered by TCGA \| \| 17 \| GBM, LGG \| Gliomas \| \| 18 \| GBM, LGG, PCPG \| Neuro-endocrine and Gliomas \| \| 19 \| KICH, KIRC, KIRP \| Kidney \| \| 20 \| KIRC, KIRP \| Kidney without KICH \| \| 21 \| LAML, PRAD, THCA, THYM \| Clustered by TCGA \| \| 22 \| LAML, THCA \| Clustered by TCGA \| \| 23 \| LUAD, LUSC \| Pulmonary without MESO \| \| 24 \| LUAD, LUSC, MESO \| Pulmonary \| \| 25 \| OV, UCEC \| Pelvic Female Reproductive \| \| 26 \| PAAD, STAD \| GI Enzyme Production \| \| 27 \| PRAD, TGCT \| Male Reproductive \| \| 28 \| SKCM, UVM \| Melanoma \| \| 29 \| UCEC, UCS \| Uterus \| |  |
| --- | --- | --- | --- | --- | --- | --- | --- | --- | --- | --- | --- | --- | --- | --- | --- | --- | --- | --- | --- | --- | --- | --- | --- | --- | --- | --- | --- | --- | --- | --- | --- | --- | --- | --- | --- | --- | --- | --- | --- | --- | --- | --- | --- | --- | --- | --- | --- | --- | --- | --- | --- | --- | --- | --- | --- | --- | --- | --- | --- | --- | --- | --- | --- | --- | --- | --- | --- | --- | --- | --- | --- | --- | --- | --- | --- | --- | --- | --- | --- | --- | --- | --- | --- | --- | --- | --- | --- | --- | --- | --- | --- |

**Supplementary Text**

**Text S1.** The final set of germline variants included in this analysis are not substantially contaminated by somatic mutations or RNA editing.

Because the final variant call set was created by merging variant calls from WXS Normal, WXS Tumor, and RNA Tumor data, we evaluated our variant calls to ensure that they were not significantly contaminated by somatic mutations or RNA editing.

The total number of somatic mutations in each patient were obtained from the TCGA Research Network [1]. <2% of the total number of variants in a patient prior to any filtering or quality control were somatic mutations (**Additional File 1: Figure S3A**). After filtering, <0.002% of germline variants in a given cancer included in this analysis caused the same base pair change as a somatic mutation (**Additional File 1: Figure S3B**). In fact, <0.02% of germline variants included in this analysis in a given cancer even overlapped in position with a somatic mutation (**Additional File 1: Figure S3C**). Therefore our final variant call set after filtering was not significantly contaminated by somatic mutations.

We next checked whether our variant call set was significantly affected by RNA editing. A set of over 2.5 million known RNA editing sites was identified from the rigorously annotated RNA editing database RADAR and overlapped with the germline variants included in this analysis [2]. <0.25% of germline variants in a given cancer included in this analysis overlapped in position with an RNA editing site (**Additional File 1: Figure S4A**).

79.6% of germline variants were called in both the WXS and RNA samples, 19.6% were called only in the WXS samples, and 0.8% were called only in the RNA samples (**Additional File 1: Figure S4B).** Because a large number of germline variants were called in both the WXS and RNA samples, we were able to evaluate the concordance between the variant calls between the WXS Normal, WXS Tumor, and RNA Tumor samples. The allele frequency of each variant in each cancer in all four variant call sets (WXS Normal, WXS Tumor, RNA Tumor, and the three variant call sets combined) was calculated and correlated with each other. The allele frequencies in the four variant call sets were very well correlated with each other (**Additional File 1: Figure S4C**), implying that the variant calls between the different samples were highly concordant. Taken together, these results suggest that somatic mutations, RNA editing, and pooling of the variant call sets did not lead to spurious germline variant calls.

Germline variant calling of all of the patients included in TCGA had previously been performed by Huang et al. [3]. We found that 93.0% of the variants called by Huang et al. were also found to have the same exact germline variant call in our analysis. For 1.5% of the variant calls there was disagreement between the two tools about whether an individual was heterozygous or homozygous for the alternate allele. 5.53% of the variants were called by GenomeVIP (Huang et al.’s tool) but not VarDict (our tool). <0.07% of the variants were called in VarDict but not GenomeVIP.

The concordance between the two germline variant call sets is quite strong, given the differences between the two studies. Huang et al. had performed variant calling on the WXS Normal samples aligned to hg19 and had performed variant calling using GenomeVIP, which integrates variant calls from Varscan, GATK, and pindel, whereas our germline variant calls were generated using VarDict from the WXS Normal, WXS Tumor, and RNA sequenced tumor samples aligned to hg38 [4-7]. Huang et al. implemented a variety of filtering criteria, including requiring an unfiltered allelic depth greater than 5 reads. We required a filtered (we excluded reads with a mapping quality less than 30 and base quality less than 25) read depth of 3 reads per sample and allele fraction of 5% The level of discordance that we found was expected, given the differences that could result from the usage of different reference genomes during alignment, filtering criteria, and variant calling tools [7].

**Text S2.** The results of our power analysis suggest that we can detect associations between germline variants with moderate to high effect sizes and patient outcome.

We evaluated our ability to detect significant associations between germline variants and patient outcome across the thirty-three cancers by calculating statistical power. The power to detect a significant association between a variant and patient outcome is dependent on multiple factors, including sample size, effect size, correlation with other covariates in the survival model, the number of patients with the germline variant, and the number of patients without the germline variant. To get a sense of our likelihood to detect associations across the thirty-three cancers at various effect sizes, we randomly sampled 10,000 germline variants from the pool of testable germline variants and calculated power for each germline variant at hazard ratios of 2, 3, 4, 5, 10, 15, and 20. The results are depicted in **Additional File 1: Figure S5**.

The results suggest that our study design would enable us to detect associations beginning around a hazard ratio of 2. With that said, our power study suggests that for every germline variant that we are able to associate with patient outcome at lower hazard ratios, we will likely fail to detect several others due to having limited statistical power for variants with lower effect sizes, even in the cancers with the largest sample sizes. Future studies with larger sample sizes will be able to detect these associations that our current study will likely miss. Furthermore, it should be noted that even if germline variants fail to be associated with patient outcome, our study is not sufficiently powered to claim that those variants are not in reality associated with outcome. Finally, the results suggest that we are extremely unlike to detect an association with germline variants with low to moderate effect sizes in ACC, CHOL, DLBC, KICH, PCPG, TGCT, THYM, UCS, and UVM.

**Text S3**. The direction (indicating whether a germline variant is associated with increased or decreased risk of poor outcome) and magnitude of the hazard ratio is correlated across cancers in which the germline variant is prognostic.

When looking at the set of variants associated with patient outcome in three or more cancers, we found that the direction of the hazard ratio for a given variant in different cancers in which it was prognostic (HR>1 implying that the variant is associated with increased risk of poor outcome or HR<1 implying that the variant is associated with decreased risk of poor outcome) was much more concordant (p<2.2E-16) than we expected based on random chance. Surprisingly, we even found the magnitude of the hazard ratio to be correlated across cancers. We identified the set of variants associated with favorable (HR<1) outcome and poor (HR>1) outcome in three or more cancers and found the hazard ratios estimated for a variant in different cancers to be correlated for both the variants associated with poor outcome (HR>1) (Spearman rho=0.146, p=5.36E-157) and variants associated with favorable outcome (HR<1) (Spearman’s rho=0.185, p=2.71E-101). Because previous studies have reported a correlation between effect size of variants identified in GWAS and allele frequency, we considered whether this correlation may be confounded by the allele frequency of these variants [8]. After controlling for allele frequency, we still find a significant partial correlation after analyzing both the variants associated with increased risk of poor outcome (Spearman rho=0.0667, p=4.024E-34) and decreased risk of poor outcome (Spearman rho=0.0584, p=2.274E-11) variants. These findings reinforce the notion that the prognostic germline variants’ effects tend to show some consistency across cancers.

**Text S4**. The alleles associated with increased risk of poor outcome of prognostic germline variants are more likely to be associated with somatic mutations in known cancer driver genes than the alleles of non-prognostic germline variants.

A previous study had identified germline variants that were associated with a significant increased incidence of somatic mutations in cancer related genes.[9] We therefore hypothesized that the prognostic variants were associated with an increased incidence of somatic mutations in driver genes in the cancer in which that variant was prognostic. To test this hypothesis, we created 353 germline variant-cancer pairs and determined the number of prognostic variants for which the allele associated with increased risk of poor outcome was associated with an increased incidence of somatic mutations relative to the protective allele. We repeated this analysis for all of the germline variants included in this analysis. We found that 47 of the 353 (13.3%) germline variant-cancer pairs were associated with an increased incidence of mutations in cancer driver genes which is more than expected by random chance (OR=1.89, p=0.0001).

**Text S5.** A detailed discussion of the twelve germline variants that cause significant amino acid changes.

To demonstrate that the prognostic germline variants identify genes that could be directly or indirectly linked to cancer progression, below we turn to the twelve germline variants in **Figure 5E** that caused substantial amino acid changes. Of these *MAP2K3* has been discussed in the main text.

*A2ML1* is a secreted protease inhibitor that inhibits all classes of proteases. When proteases cleave the central bait domain of *A2ML1*, conformational changes cause an internal thiol ester, formed by C970 and Gln973, to become highly reactive. This thiol ester bond binds the protease and facilitates the formation of covalent bonds between *A2ML1* and the protease, resulting in protease entrapment and inhibition [10]. In our analysis, the germline variant rs1558526 was associated with favorable patient outcome in ovarian cancer patients and resulted in a C970Y change in *A2ML1*. Because the very cysteine residue that forms the internal thiol ester is lost, this amino acid change likely disrupts *A2ML1’*s protease inhibition function (**Additional File 1: Figure S7A**). This result suggests that certain extracellular proteases which *A2ML1* may normally inhibit may have anti-tumor effects, for example by degrading angiogenic factors or anti-immune factors.

*CRYBG1/AIM1* (absent in melanoma) is a protein that localizes to the cytoskeleton. Loss of *CRYBG1* in prostate cancer cells leads to increased G-actin (relative to F-actin), cell migration, invasion and soft agar colony formation. Binding of *AIM1* to actin requires the six C terminal domains made of 12 βγ crystallin motifs [11]. We found rs6174114 in *CRYBG1* to be associated with poor patient outcome in pancreatic cancer. This variant changes L1235 to P in the fifth domain of *CRYBG1*. Substitution of proline at this position could disrupt the packing of the beta sheets that make a β or γ motif (**Additional File 1: Figure S7B**), resulting in loss of *CRYBG1* function and therefore increase cell migration, invasion, and soft agar colony formation. This would explain the poor patient outcome associated with this germline variant. Somatic mutation or epigenetic suppression of *CRYBG1* has been seen in melanomas, lymphomas, and prostate carcinoma. Decreased expression of the protein associated with metastasis [11].

*EIF2AK4/GCN2* is a protein kinase that is activated under stress by binding to uncharged tRNAs through its histidyl-tRNA-synthetase domain. This kinase is important for decreasing protein translation and for activating specific translation of genes like *ATF4* and *p21/CDKN1A* under conditions of stress often seen inside tumors like amino acid starvation and glucose starvation. We found the germline variant rs35602605 in *EIF2AK4* to be associated with poor prognosis. This variant causes a G1306S amino acid change in the histidyl-tRNA synthetase-like domain (**Additional File 1: Figure S7C**). This variant may disrupt the ability of the histidyl-tRNA synthetase-like domain to bind uncharged tRNAs and thereby protect the cancer cells from translation of stress-induced genes like *CDKN1A* that restrain tumor proliferation. If true, this would explain the association of this germline variant with poor patient outcome.

The other gene-products identified by prognostic variants in **Figure 5E** also warrant a detailed examination. Two of them could be important for immune response to a tumor. *FCRL6* binds to MHC class II proteins and acts as an immune checkpoint protein that is often upregulated in Tumor infiltrating lymphocytes [12]. It is particularly interesting that *FCRL6* expression of T lymphocytes is decreased five-fold in acute and chronic myeloid leukemias because the rs61823162 variant (which truncates the protein) is associated with outcome in LAML [13]. *EPHA10* is a non-functional tyrosine kinase receptor for ephrins. The G749E mutation is located in the tyrosine kinase domain, which upregulates PD-L1 protein expression [14]. Three genes are involved in intracellular vesicle transport, membrane fusion and cell migration: *BORCS5* recruits the *ARL8B* GTPase to lysosomes for lysosomal movement and function, *KDELR3* is involved in retaining proteins in the endoplasmic reticulum, and *MYOF* facilitates vesicle fusion. Two are involved in GPCR pathways: *OR10X1 is* an olfactory receptor and *SAG/arrestin1* binds to GPCRs (such as rhodopsin) to terminate signaling. Many olfactory receptors are ectopically expressed in several cancer and their activation decreases cancer cell proliferation and migration and increases apoptosis [15, 16]. The I-76 of *SAG* that is altered by the variation is located in the highly conserved finger loop of motif 2, (E/D)x(I/L)xxxGL, which is extended and buried in the rhodopsin (GPCR)-SAG interface [17]. Finally *ECD/SGT1* associates with many cellular proteins relevant for cancer, *MDM2*, *Rb*, *HSP90*, *SKP1*, and *RUVBL1*, the last in particular using the C-terminal region of *ECD* that is mutated in the prognostic variant.

**Supplementary References**

1. Ellrott K, Bailey MH, Saksena G, Covington KR, Kandoth C, Stewart C, Hess J, Ma S, Chiotti KE, McLellan M, et al: **Scalable Open Science Approach for Mutation Calling of Tumor Exomes Using Multiple Genomic Pipelines.** *Cell Syst* 2018, **6:**271-281.e277.

2. Ramaswami G, Li JB: **RADAR: a rigorously annotated database of A-to-I RNA editing.** *Nucleic Acids Res* 2014, **42:**D109-113.

3. Huang KL, Mashl RJ, Wu Y, Ritter DI, Wang J, Oh C, Paczkowska M, Reynolds S, Wyczalkowski MA, Oak N, et al: **Pathogenic Germline Variants in 10,389 Adult Cancers.** *Cell* 2018, **173:**355-370.e314.

4. McKenna A, Hanna M, Banks E, Sivachenko A, Cibulskis K, Kernytsky A, Garimella K, Altshuler D, Gabriel S, Daly M, DePristo MA: **The Genome Analysis Toolkit: a MapReduce framework for analyzing next-generation DNA sequencing data.** *Genome Res* 2010, **20:**1297-1303.

5. DePristo MA, Banks E, Poplin R, Garimella KV, Maguire JR, Hartl C, Philippakis AA, del Angel G, Rivas MA, Hanna M, et al: **A framework for variation discovery and genotyping using next-generation DNA sequencing data.** *Nat Genet* 2011, **43:**491-498.

6. Van der Auwera GA, Carneiro MO, Hartl C, Poplin R, Del Angel G, Levy-Moonshine A, Jordan T, Shakir K, Roazen D, Thibault J, et al: **From FastQ data to high confidence variant calls: the Genome Analysis Toolkit best practices pipeline.** *Curr Protoc Bioinformatics* 2013, **43:**11.10.11-33.

7. Lai Z, Markovets A, Ahdesmaki M, Chapman B, Hofmann O, McEwen R, Johnson J, Dougherty B, Barrett JC, Dry JR: **VarDict: a novel and versatile variant caller for next-generation sequencing in cancer research.** *Nucleic Acids Res* 2016, **44:**e108.

8. Park JH, Gail MH, Weinberg CR, Carroll RJ, Chung CC, Wang Z, Chanock SJ, Fraumeni JF, Jr., Chatterjee N: **Distribution of allele frequencies and effect sizes and their interrelationships for common genetic susceptibility variants.** *Proc Natl Acad Sci U S A* 2011, **108:**18026-18031.

9. Carter H, Marty R, Hofree M, Gross AM, Jensen J, Fisch KM, Wu X, DeBoever C, Van Nostrand EL, Song Y, et al: **Interaction Landscape of Inherited Polymorphisms with Somatic Events in Cancer.** *Cancer Discov* 2017, **7:**410-423.

10. Galliano MF, Toulza E, Gallinaro H, Jonca N, Ishida-Yamamoto A, Serre G, Guerrin M: **A novel protease inhibitor of the alpha2-macroglobulin family expressed in the human epidermis.** *J Biol Chem* 2006, **281:**5780-5789.

11. Haffner MC, Esopi DM, Chaux A, Gurel M, Ghosh S, Vaghasia AM, Tsai H, Kim K, Castagna N, Lam H, et al: **AIM1 is an actin-binding protein that suppresses cell migration and micrometastatic dissemination.** *Nat Commun* 2017, **8:**142.

12. Johnson DB, Nixon MJ, Wang Y, Wang DY, Castellanos E, Estrada MV, Ericsson-Gonzalez PI, Cote CH, Salgado R, Sanchez V, et al: **Tumor-specific MHC-II expression drives a unique pattern of resistance to immunotherapy via LAG-3/FCRL6 engagement.** *JCI Insight* 2018, **3**.

13. Kulemzin SV, Zamoshnikova AY, Yurchenko MY, Vitak NY, Najakshin AM, Fayngerts SA, Chikaev NA, Reshetnikova ES, Kashirina NM, Peclo MM, et al: **FCRL6 receptor: expression and associated proteins.** *Immunol Lett* 2011, **134:**174-182.

14. Yang WH, Cha JH, Xia W, Lee HH, Chan LC, Wang YN, Hsu JL, Ren G, Hung MC: **Juxtacrine Signaling Inhibits Antitumor Immunity by Upregulating PD-L1 Expression.** *Cancer Res* 2018, **78:**3761-3768.

15. Weber L, Al-Refae K, Ebbert J, Jagers P, Altmuller J, Becker C, Hahn S, Gisselmann G, Hatt H: **Activation of odorant receptor in colorectal cancer cells leads to inhibition of cell proliferation and apoptosis.** *PLoS One* 2017, **12:**e0172491.

16. Ranzani M, Iyer V, Ibarra-Soria X, Del Castillo Velasco-Herrera M, Garnett M, Logan D, Adams DJ: **Revisiting olfactory receptors as putative drivers of cancer.** *Wellcome Open Res* 2017, **2:**9.

17. Peterson YK, Luttrell LM: **The Diverse Roles of Arrestin Scaffolds in G Protein-Coupled Receptor Signaling.** *Pharmacol Rev* 2017, **69:**256-297.
